# Aquatic Ecosystem Response to Timber Harvesting for the Purpose of Restoring Aspen

**DOI:** 10.1371/journal.pone.0084561

**Published:** 2013-12-20

**Authors:** Bobette E. Jones, Monika Krupa, Kenneth W. Tate

**Affiliations:** 1 Lassen National Forest, United States Department of Agriculture Forest Service, Susanville, California, United States of America; 2 Department of Plant Sciences, University of California Davis, Davis, California, United States of America; Brigham Young University, United States of America

## Abstract

The removal of conifers through commercial timber harvesting has been successful in restoring aspen, however many aspen stands are located near streams, and there are concerns about potential aquatic ecosystem impairment. We examined the effects of management-scale conifer removal from aspen stands located adjacent to streams on water quality, solar radiation, canopy cover, temperature, aquatic macroinvertebrates, and soil moisture. This 8-year study (2003–2010) involved two projects located in Lassen National Forest. The Pine-Bogard Project consisted of three treatments adjacent to Pine and Bogard Creeks: (i) Phase 1 in January 2004, (ii) Phase 2 in August 2005, and (iii) Phase 3 in January 2008. The Bailey Project consisted of one treatment adjacent to Bailey Creek in September 2006. Treatments involved whole tree removal using track-laying harvesters and rubber tire skidders. More than 80% of all samples analyzed for NO_3_-N, NH_4_-N, and PO_4_-P at Pine, Bogard, and Bailey Creeks were below the detection limit, with the exception of naturally elevated PO_4_-P in Bogard Creek. All nutrient concentrations (NO_3_-N, NH_4_-N, PO_4_-P, K, and SO_4_-S) showed little variation within streams and across years. Turbidity and TSS exhibited annual variation, but there was no significant increase in the difference between upstream and downstream turbidity and TSS levels. There was a significant decrease in stream canopy cover and increase in the potential fraction of solar radiation reaching the streams in response to the Pine-Bogard Phase 3 and Bailey treatments; however, there was no corresponding increase in stream temperatures. Macroinvertebrate metrics indicated healthy aquatic ecosystem conditions throughout the course of the study. Lastly, the removal of vegetation significantly increased soil moisture in treated stands relative to untreated stands. These results indicate that, with careful planning and implementation of site-specific best management practices, conifer removal to restore aspen stands can be conducted without degrading aquatic ecosystems.

## Introduction

Trembling aspen (*Populus tremuloides* Michx.) occurs throughout North America, including the montane zone of California’s Sierra Nevada and southern Cascade ranges [1]. Aspen is considered a keystone species providing crucial habitat to support a high diversity of local and landscape plant species [2], bird communities [3-5], mammals [6], and insects and other invertebrates [7]. Additionally, aspen stands provide important ecosystem services such as increased water yields and soil moisture [8], and act as natural firebreaks [9,10].

Declines in the health and distribution of aspen stands across western North America have been observed over the past century to the present day [11-14]. Much of this decline is attributable to conifer encroachment stimulated by the absence of natural fire regimes, as well as historic and current heavy browsing of aspen suckers by domestic and native herbivores [15-17]. Aspen is a clonal species and disturbance stimulates its vegetative reproduction [18]. When fire return intervals are lengthened, the disturbance of aspen and their resultant vegetative reproduction is reduced [11,19]. Additionally, fire suppression places aspen at a competitive disadvantage to shade-tolerant conifers, as aspen require sunlight and warm soil for successful regeneration [13,20,21]. The results of an aspen inventory conducted from 2000 - 2011 that assessed the current status and risk of loss of 700 live aspen stands (approximately 99 % of known stands) totaling 1,540 ha on the Eagle Lake Ranger District, Lassen National Forest, documented that 79 % of stands were at high risk of being lost. At least 45 known stands have expired with no living aspen present and no means of recruitment. Conifer encroachment was the major risk factor associated with 96 % of inventoried stands [18]. 

Recent studies have found that the use of commercial timber harvest techniques to remove conifers is an effective treatment for stimulating aspen regeneration [10,22,23], and that long-term success of aspen stand restoration can be achieved when this treatment is coupled with management of ungulate herbivory [16,24]. The broad-scale implementation of silvicultural treatments in this region is of concern however, because a significant number of conifer encroached aspen stands are associated with streams [18]. Timber harvest activities that occur adjacent to rivers and streams have the potential to affect soil moisture dynamics [25,26], water quality [27-33], stream temperature through increased solar radiation inputs [34-36], and aquatic communities [37-39]. However, conifer encroached aspen stands that are not released will expire and overall landscape habitat complexity and biodiversity will continue to decline. Previous studies have found that the implementation of best management practices (BMPs) during timber harvest can prevent or limit aquatic ecosystem degradation [40-42]. Such studies also indicate that, when BMPs are followed, any changes in the aquatic ecosystem that have occurred often return to pre-harvest conditions within 5 years [42-45]. 

**Figure 1:**
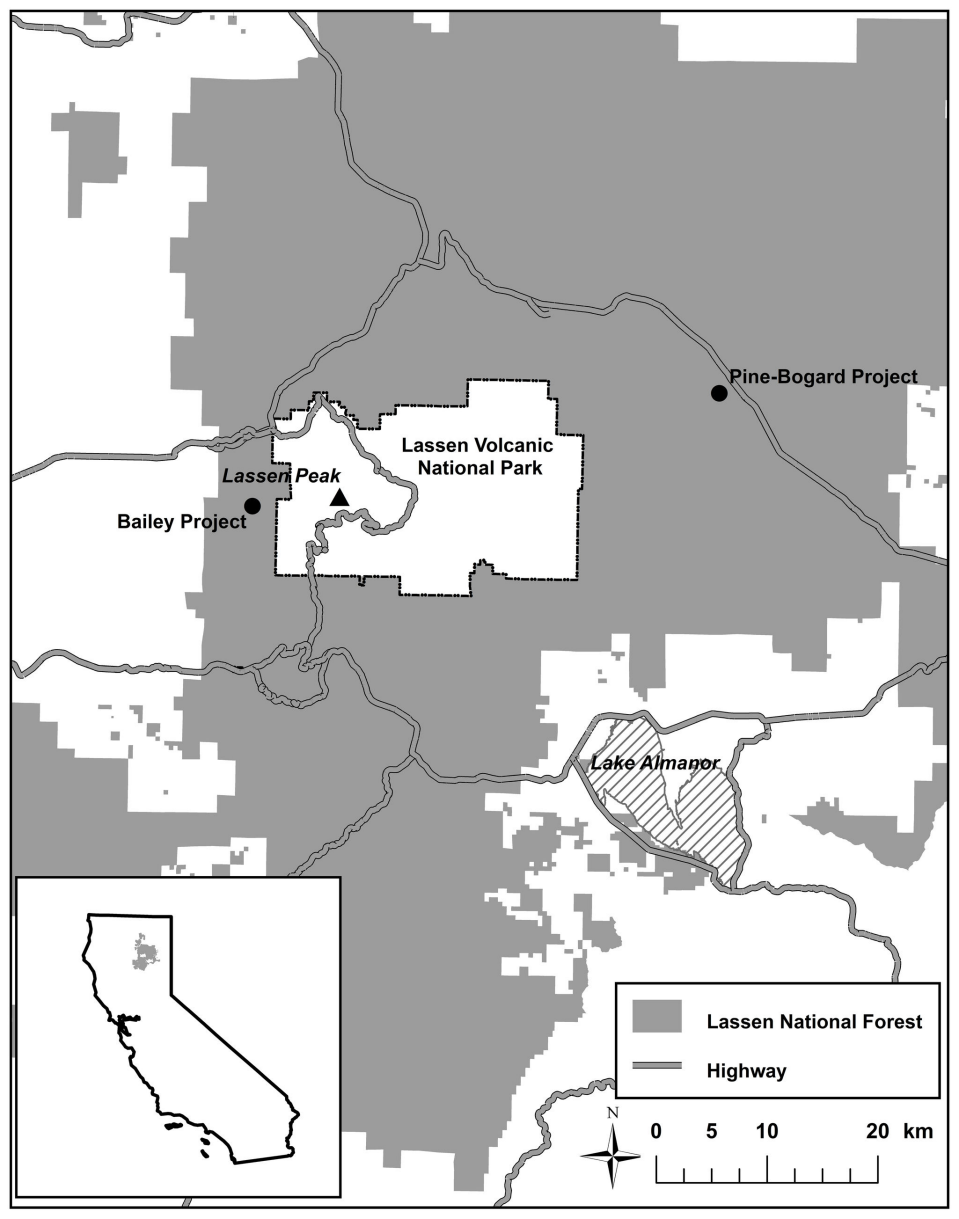
Pine-Bogard Project and Bailey Project locations.

The purpose of this study was to quantify the occurrence and magnitude of the impacts of management-scale prescriptive conifer removal through commercial timber harvest on aquatic habitat quality in streams adjacent to the conifer removal activities and on soil moisture dynamics within the treated aspen stands. To this end, we performed water quality, stream temperature, solar radiation, canopy cover, aquatic macroinvertebrate, and soil moisture tension monitoring, both before and after the implementation of each of four conifer removal treatments in Lassen National Forest (Figure 1). We hypothesized that conifer removal would cause: (1) an increase in nutrient concentrations, total suspended solids, and turbidity levels, (2) an increase in stream temperatures as a result of decreased canopy cover allowing more solar radiation to reach the streams, (3) a decrease in aquatic ecosystem health as indicated by macroinvertebrate metrics, and (4) an increase in soil moisture. 

## Materials and Methods

### 2.1: Ethics statement

This field study was conducted in collaboration with the USDA Forest Service, and so all permissions for site access were granted and no permits were required. An Environmental Impact Statement addressing the timber harvest treatments was completed by the USDA Forest Service in accordance with National Environmental Policy Act requirements. Macroinvertebrate sampling was performed following approved protocols described by Barbour et al. [46].

### 2.2: Study Area

This study was carried out at two locations: (1) the confluence of Pine and Bogard Creeks (Bogard Creek is a tributary to Pine Creek) (40°34’42.66” N, 121°05’49.18” W) in the Eagle Lake Ranger District, which is located on the eastern side of Lassen National Forest at an elevation of approximately 1,740 m, and (2) at the South Fork of Bailey Creek (40°28’46.48” N, 121°35’45.84” W) in the Hat Creek Ranger District, which is located on the western side of Lassen National Forest at an elevation of approximately 1,920 m. The Pine Creek and Bailey Creek watersheds have total surface areas of approximately 591 and 154 km^2^, respectively.

Vegetation in the Pine and Bogard Creek study area is dominated by Ponderosa pine (*Pinus ponderosa* Laws.), sagebrush (*Artemisia tridentata* Nutt.), and aspen. The area has slopes from 0 to 4 %, with a northeast aspect at the uppermost reaches, and level at the valley floor. Geology is dominated by basalt. Soils in the valley floor are dominated by loamy-skeletal, mixed, frigid Ultic Haploxerolls, and soils in the upper slopes are dominated by fine-loamy, mixed, frigid Pachic Ultic Argixerolls. Both Pine and Bogard Creeks are spring fed, but receive a substantial amount of snowmelt, particularly in May and June. Pine Creek has a much larger watershed area than Bogard Creek, and as a result, a larger fraction of its water is derived from snowmelt. From the months of May through September, the average width of Bogard Creek along the stream reach that was monitored in this study was approximately 1 m, and the average width of Pine Creek was approximately 3 m. During summer months, Pine Creek becomes intermittent approximately 1,500 m below its confluence with Bogard Creek. The Mediterranean climate consists of dry, warm summers, and wet, cool winters. Precipitation primarily occurs as snowfall from November through May [47]. Based on models from the PRISM Climate Group, long-term average annual precipitation is 630 mm, and ranged from 460 to 1,070 mm during the study period [48]. The primary land uses in the area are recreation (camping, hunting, snowmobiling), cattle grazing, which occurs annually from approximately June 1 through September 30, and vegetation management. 

Vegetation in the Bailey Creek study area is dominated by perennial meadow herbaceous species, Lodgepole pine (*Pinus contorta* Dougl.),White fir (*Abies concolor* (Gordon & Glend) Lindley), Willow (*Salix lucida* Muhl. ssp. *lasiandra* (Benth.) E. Murray), and aspen. The area has slopes from 0 to 4 %. The geology is typified by moraine complexes with well-preserved morphology and a weakly oxidized soil zone approximately 50 cm thick. Soils are dominated by Aquolls. Bailey Creek is spring fed but receives a substantial amount of snowmelt, particularly in May and June. From the months of May through September, the average width of Bailey Creek along the stream reach that was monitored in this study was approximately 4 m. Similarly to the Pine and Bogard Creek study area, the climate is Mediterranean and precipitation primarily occurs as snowfall from November through May [47]. Based on models from the PRISM Climate Group, long-term average annual precipitation is 1,590 mm, and ranged from 810 to 1,870 mm during the study period [48]. The primary land uses in the area are recreation (camping, fishing, hunting) and vegetation management. Historically the area was grazed by livestock, but has not been grazed for more than 30 years. 

### 2.3: Project Design

**Figure 2:**
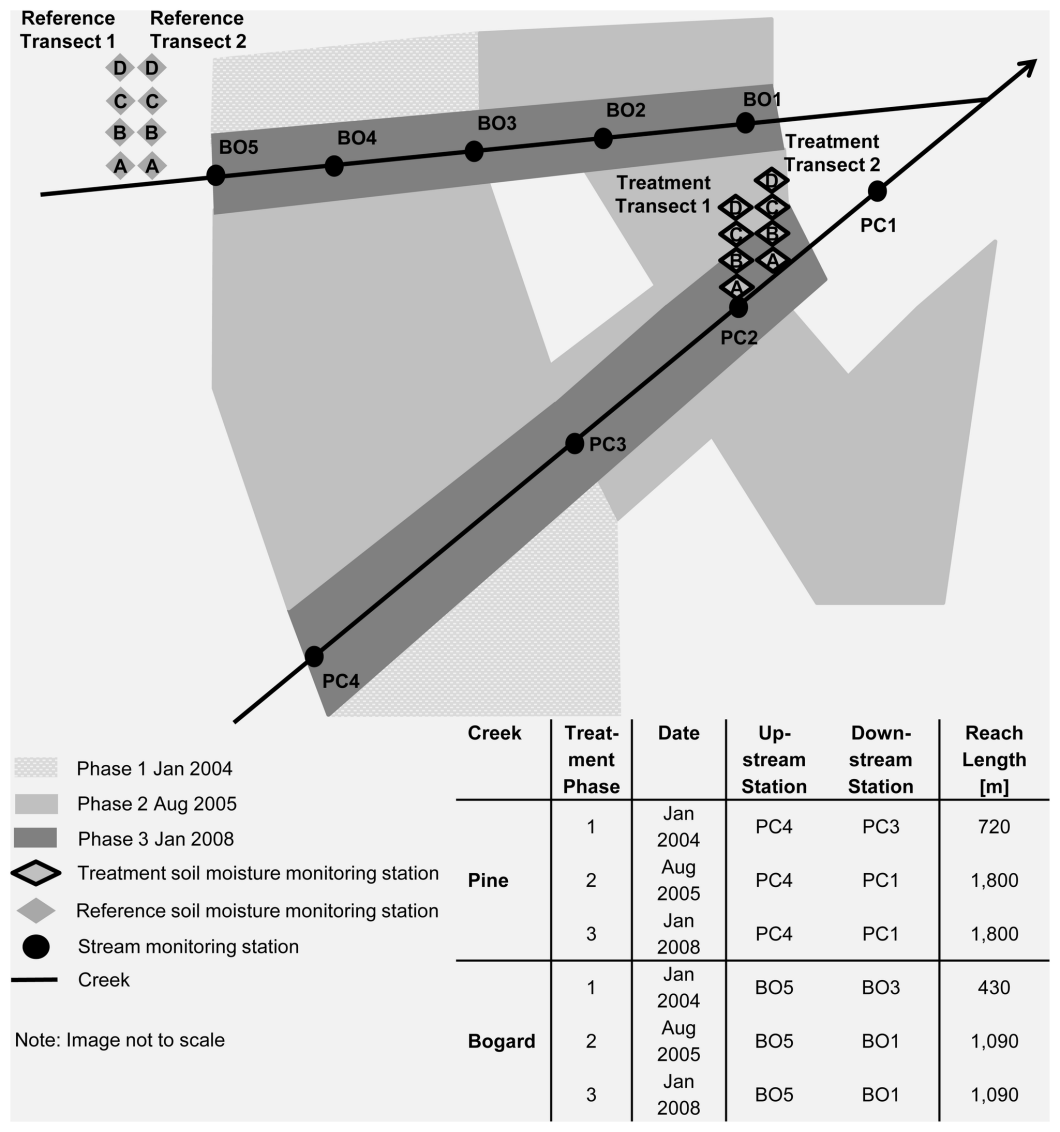
Illustration of Pine-Bogard Project Phases 1-3 treatment areas and stream and soil monitoring station locations.

**Figure 3:**
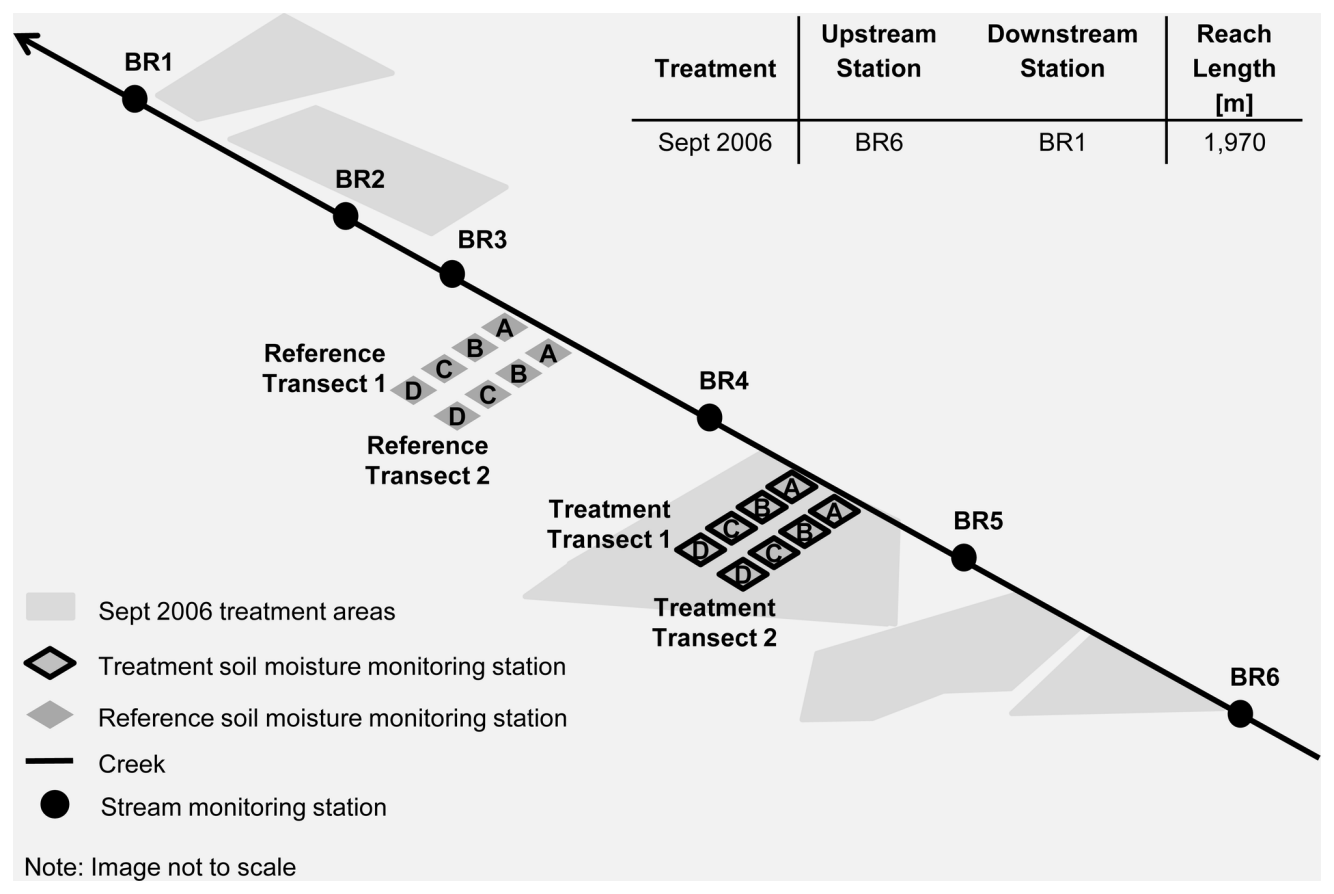
Illustration of Bailey Project treatment areas and stream and soil monitoring station locations.

This study involved two different management-scale conifer removal projects (Figures 2 and 3). The Pine-Bogard Project consisted of conifer removal treatments in three phases: (i) Phase 1 in January 2004, (ii) Phase 2 in August 2005, and (iii) Phase 3 in January 2008. The Bailey Project consisted of conifer removal at Bailey Creek, which was implemented as one treatment in September 2006. Both project areas were chosen for restoration because the aspen stands were at high risk of loss due to extensive conifer encroachment.

The following parameters were measured in order to understand the impacts of management-scale conifer removal on aquatic ecosystems and soil moisture dynamics: (i) soil moisture tension, (ii) stream and air temperature, (iii) stream discharge and water quality, including nitrate as N (NO_3_-N), ammonium as N (NH_4_-N), phosphate as P (PO_4_-P), sulfate as S (SO_4_-S), potassium (K), total suspended solids (TSS), and turbidity, (iv) stream canopy cover and solar radiation inputs, and (v) aquatic macroinvertebrates. Soil moisture was investigated through the establishment of discrete soil moisture tension monitoring stations along transects located within treatment and reference aspen stands (Figures 2 and 3). Treatment stands are conifer encroached aspen stands in which conifer removal took place, while reference stands are untreated conifer encroached aspen stands. Streamwater quality, discharge, temperature, and aquatic macroinvertebrates, were measured at stream monitoring stations. Each stream included at least one station immediately upstream and one station immediately downstream of the treatment stands (Figures 2 and 3). Canopy cover and solar radiation measurements were made along the stream reaches located between the upstream and downstream stations for each treatment (refer to tables in Figures 2 and 3).

#### 2.3.1: Conifer Removal Implementation

Pine-Bogard Phase 1 treatment occurred in January of 2004 between Pine Creek stations PC3 and PC4 and Bogard Creek stations BO3 and BO5 (Figure 2). The total treatment area for this phase was approximately 24 ha, with 720 m of Pine Creek and 430 m of Bogard Creek affected by the treatment. Timber harvest was implemented over snow with a minimum requirement of 60 cm of snow or 10 cm of frozen ground. Whole tree removal using track-laying harvesters and rubber tire skidders was used to remove conifers more than 25 m from stream. Whole tree removal is the standard practice used because it creates less slash, and over snow was used to protect the soil surface. Trees within 25 m of stream were hand felled and end-lined to reduce compaction and sedimentation within areas closest to the streams. Typically, all conifers less than 75 cm diameter at breast height (dbh) were removed, except for conifers directly contributing to streambank stability or other site-specific benefits. Hand-felling of conifers less than 30 cm dbh occurred post-harvest. The treatment area before and after Phase 1 conifer removal is illustrated in Photos S1, S2, and S3.

Pine-Bogard Phase 2 treatment was designed to maximize the amount of dry season harvest in order to avoid the excessive slash that was observed during Phase 1 over snow harvest. Phase 2 treatment occurred from August 10, 2005 through September 12, 2005 between Pine Creek stations PC1 and PC4 and Bogard Creek stations BO1 and BO5 (Figure 2). The total treatment area for this phase was approximately 80 ha, with 1,800 m of Pine Creek and 1,090 m of Bogard Creek affected by the treatment. Whole tree removal using track-laying harvesters and rubber tire skidders was used to remove conifers within 4 to 40 m from stream. This distance was identified on the ground using site specific factors including slope and ground cover. Photo S4 illustrates an area where conifer removal took place during Phase 2 treatment adjacent to an untreated area.

Pine-Bogard Phase 3 treatment was designed to treat the remaining areas adjacent to streams that could not be accessed during Phase 2 treatment. The treatment occurred in January 2008 between Pine Creek stations PC1 and PC4 and Bogard Creek stations BO1 and BO5 (Figure 2). The total treatment area for this phase was approximately 13 ha, with 1,800 m of Pine Creek and 1,090 m of Bogard Creek affected by the treatment. Timber harvest was implemented over snow with a minimum requirement of 60 cm of snow or 10 cm of frozen ground. Whole tree removal using track-laying harvesters and rubber tire skidders was used. A mechanical equipment boundary was delineated from the water’s edge to the area with complete and continuous vegetation. No equipment was allowed to enter this area but conifers that did not contribute to streambank stability and were less than 75 cm dbh were removed through hand felling or reaching with a mechanical harvester boom. The treatment area before and after Phase 3 conifer removal is illustrated in Photos S5, S6, and S7.

A dry-season conifer removal project occurred in September 2006 between stations BR1 and BR6 along Bailey Creek (Figure 3). The total treatment area for this project was approximately 4.5 ha, with 560 m of the total BR1 to BR6 reach length (1,970 m) affected by the treatment. Whole tree removal using track-laying harvesters and rubber tire skidders was used between 1.5 to 90 m from stream depending upon slope and ground cover. Skid trails were designated in areas that did not contain riparian vegetation. Typically, all conifers 10 to 75 cm dbh were removed except for conifers located within 8 meters of the stream and contributing to stream bank stability. Conifers less than 50 cm dbh were hand felled. All conifers 10 cm dbh or smaller were cut, hand piled and burned outside of the aspen clone root zone to prevent damage to the root system. The treatment area before and after conifer removal is illustrated in Photos S8, S9, and S10.

#### 2.3.2: Stream discharge and water quality

Stream water samples were grab sampled every 2 weeks at each stream monitoring station. Pine-Bogard samples were collected from 2003 through 2010. Bailey samples were collected from 2003 through 2004, and from 2006 through 2010. Sampling was focused on both the peak flow period, which occurs in late May/early June and is the period of most rapid snowmelt, and the summer base flow period. Sampling began as early as May 19 and ended as late as October 12 at Pine and Bogard Creek stations, and began as early as June 3 and ended as late as October 7 at Bailey Creek stations. Sampling did not occur from about October to May because the streams are largely frozen and flows are low. The sampling periods for this study are similar to that of other studies performed in areas where much of the year is characterized by precipitation falling as snow and by freezing temperatures [28,32,33,44]. Historic stream discharge data from USGS Station 10359250 (1961-1978), which was located on Pine Creek near sampling location PC1 (Figure 2), indicates that the May through September sampling period generally captured at least 80 percent of annual discharge at Pine and Bogard Creeks. Readings started relatively later in the year at the Bailey Creek study area because it is located at a higher elevation than the Pine-Bogard study area and consequently snow melt occurs approximately 1 month later. 

Stream discharge was measured at the same time as grab samples were collected using the area-velocity method. With this method, discharge was calculated as: mean velocity × stream width × mean stream depth [49]. A minimum of 3 stream depth readings were taken across each stream cross-section – at thalawag and at mid-point between thalawag and each streambank. Water velocity was measured at each of these three depth reading locations. A Global Waters flow meter (Global Waters Inc., Gold River, California, USA) was used to estimate velocity whenever water depth was greater than 10 cm. The float method was used whenever water depth was < 10 cm and thus could not accommodate the flow meter [49]. Velocity meter readings were collected at 0.6 of stream depth. A correction factor of 0.85 was used to adjust surface velocities when the float method was used.

Grab samples were refrigerated (4 °C) and transported to UC Davis for analysis. Nephelometric turbidity units (ntu) and pH were measured on non-filtered subsamples using standard methods SM2130 and SM4500-H^+^, respectively [50]. To measure TSS, a 170 mL subsample was passed through a 0.45 µm membrane filter. The filters were dried in a desiccator until a stable weight was achieved (typically 5 to 7 days). The filter mass was measured on an analytical balance accurate to 0.001 g. The levels of TSS were then determined as the change in mass of the filter before and after filtration of the known volume of water. The concentrations of NO_3_-N, NH_4_-N, PO_4_-P, K, and SO_4_-S were measured using ion chromatography (Dionex 500x; CS12 cations; AS4A anions) on subsamples filtered through a 0.45 µm membrane filter.

#### 2.3.3: Stream and air temperature

Stream temperature data was collected at all monitoring stations on Pine, Bogard, and Bailey Creeks (Figures 2 and 3) using Onset Optic StowAway temperature loggers. Additionally, air temperature data was collected using Onset Optic StowAway temperature loggers at one location in the Pine-Bogard Project area, and at one location at the Bailey Project area. All loggers were set to record temperature every 0.5 hours. Pine-Bogard temperature data was collected from 2003 through 2010. Bailey temperature data was collected from 2003 through 2004, and from 2006 through 2010. Loggers were deployed as early as May 19 and retrieved as late as October 12 at Pine and Bogard Creek stations, and deployed as early as June 3 and retrieved as late as October 7 at Bailey Creek stations. Daily maximum and average temperatures, as well as 7-day running average maximum and average temperatures were calculated using the recorded data.

#### 2.3.4: Canopy cover and solar radiation

Percent stream canopy cover was measured with a convex spherical densiometer and represents the amount of sky above a point on the stream channel which is blocked from view by vegetation. At each sample point along a given stream reach, measurements were made facing downstream, right bank, upstream, and left bank, and then the average of these values was calculated [51]. 

The potential fraction of solar radiation reaching each stream was estimated based on measurements collected with a solar pathfinder (Solar Pathways, Hartford, South Dakota). Solar pathfinder measurements integrate the effects of the sun’s path, vegetative canopy cover, topographic shading, and stream channel aspect to estimate the potential fraction of available solar radiation (0 to 100%) reaching a site at a given latitude for each month of the year. Solar pathfinder measurements can overestimate the quantity of solar radiation reaching a site because they do not take cloud cover and atmospheric turbidity into account. A detailed description of solar pathfinders can be found in Platts et al. [52]. We concern ourselves with the months of May through September, which represent the warmest period in the region, when elevated stream water temperatures might be of concern.

There was no canopy cover or solar radiation data collected for Pine-Bogard Phase 1. Pine-Bogard Phase 2 canopy cover and solar radiation measurements were taken at the stream reach between stations BO1 and BO5, and at the stream reach between stations PC1 and PC4, before (June 2005) and after (September 2005) treatment implementation. Following Pine-Bogard Phase 3 treatment implementation, canopy cover and solar radiation measurements were taken in June 2008 at the stream reach between stations BO1 and BO5, and at the stream reach between stations PC1 and PC4. These measurements were compared to the September 2005 measurements taken following Pine-Bogard Phase 2 treatment. Measurements along Pine and Bogard Creeks were taken every 40 m during each data collection event (n= 45 and 28 for Pine and Bogard Creeks, respectively). Bailey Project canopy cover and solar radiation readings were taken at the stream reach between stations BR1 and BR6, before (September 2003) and after (July 2007) treatment implementation. Measurements along Bailey Creek were taken every 55 m (n= 36).

#### 2.3.5: Aquatic macroinvertebrates

Stream macroinvertebrate collections were made at midstream (BO3) and upstream (BO5) stations along Bogard Creek, at midstream (PC3) and upstream (PC4) stations along Pine Creek, and at midstream (BR4) and upstream (BR6) stations along Bailey Creek in June-July of 2003, 2004, 2007, 2008, and 2010. Samples were collected according to the single habitat approach for riffles described by Barbour et al. [46]. At each sample station, two sub-samples were collected along a transect perpendicular to streamflow (center-right and center-left from banks) and composited as 1 sample for analysis. Transects were established across riffle areas. All sub-samples were collected with a D-ring kick net (500 micron mesh) from a sample area of 0.09 m^2^ for a sample time of 3 minutes per sample. Collections were immediately stabilized with 95 % ethanol. Taxonomic analysis was conducted at the BLM BugLab on the campus of Utah State University following the methods described by Moulton et al. [53] and Cuffney et al. [54]. Samples were taxonomically analyzed to genus and species where possible and standard metrics describing macroinvertebrate assemblage characteristics were calculated from the raw taxa data.

#### 2.3.6: Soil Moisture

Soil moisture, measured as soil moisture tension in centibars, was investigated using permanently established gypsum blocks [55]. Higher values of soil moisture tension indicate drier soil conditions. Soil moisture tension measurements were taken approximately every 2 weeks from as early as May 19 to as late as October 12 from 2003 through 2010 at Pine and Bogard Creeks, and from as early as June 3 to as late as October 7 in 2004, and from 2006 through 2010, at Bailey Creek. Soil moisture tension monitoring stations were located in areas that provided a representative sample of the whole stand, excluding skid trails and landings.

At Pine and Bogard Creeks, 2 transects of 4 monitoring stations were established from near stream to uplands in the treatment area, and in a nearby reference area, in order to capture a gradient of soil moisture conditions, as illustrated in Figure 2. Gypsum blocks were permanently established at 15 and 45 cm in depth at each monitoring station (n = 16 at each depth, 8 treatment, 8 reference). These depths were chosen because they correspond to the bottom and mid-depths of herbaceous plant root mass in the Pine-Bogard and Bailey project areas. Phase 1 treatment did not affect the treatment transects. Phase 2 treatment affected stations C and D in both treatment transects, which were then compared to stations C and D in the reference transects (Figure 2). Phase 3 treatment affected stations A and B in both treatment transects, which were then compared to stations A and B in the reference transects (Figure 2). 

At Bailey Creek, 2 transects of 5 monitoring stations were established from near stream to uplands in the treatment area, and in a nearby reference area, as illustrated in Figure 3. Gypsum blocks were permanently established at 15 and 45 cm in depth at each monitoring station (n = 20 at each depth, 10 treatment, 10 reference).

#### 2.3.7: Data Analysis

Statistical analysis was designed to test the hypotheses that the various aquatic ecosystem parameters we measured were degraded (i.e. increased nutrient concentrations, decreased macroinvertebrate diversity) following conifer removal treatments. We used linear mixed effects (LME) and generalized linear mixed effects (GLMM) analyses to determine the occurrence and magnitude of change in each aquatic ecosystem parameter between treated/downstream and reference/upstream sample locations before versus after treatment [56-58]. A separate analysis was conducted for each parameter. The specific parameters analyzed were: NO_3_-N, NH_4_-N, PO_4_-P, K, SO_4_-S, turbidity, TSS, canopy cover, solar radiation, stream temperature, macroinvertebrate metrics (% Tolerant, % Intolerant , Shannon Diversity Index, number of families), and soil moisture tension. For soil moisture tension, separate mixed effects models were used for the 15 and 45 cm depths at (i) the Bailey transects, (ii) stations A and B of the Pine-Bogard transects, and (iii) stations C and D of the Pine-Bogard transects. The fixed effects variables in each mixed effects model were Location (downstream vs upstream; or treated vs reference area), Time (before vs after conifer removal), and the interaction of Location and Time (Time x Location). A significant interaction would indicate the presence of a treatment effect. Because there was no reference area for canopy cover and solar radiation, the mixed effects models for these parameters included only the Time variable. The two random effects variables in each mixed effects model were Sample Unit and Year, as crossed random effects. Sample Unit was used as a random term in order to account for the autocorrelation introduced by repeated measures made at each stream monitoring location or soil moisture tension sensor. Year was used as a random term to account for the annual variation in the parameters driven by environmental factors that vary across years and can be expected to affect the variables similarly across all stations (e.g. temperature and precipitation), but which were not of interest to this study. LMEs (normal distribution, homogenous variance) were used for solar radiation, canopy cover, stream temperature, and soil moisture tension analysis. GLMMs (Poisson distribution) were used for all other parameters. Conformity to the assumptions associated with these analyses was confirmed with standard diagnostic tests and graphs. All analyses were conducted with Stata/SE software [59].

## Results and Discussion

### 3.1: Water Quality

The modification of nutrient concentrations in streams through commercial timber harvest activities can alter aquatic ecosystem structure and function [34,43,45]. In particular, the levels of NO_3_-N, NH_4_-N, and PO_4_-P are of concern because increases in these nutrients have been found to cause eutrophication [60-62]. Additionally, increases of sediment levels in streams as a result of timber harvest activities have been found to modify streambed surface conditions, decrease light penetration, and alter primary production, which can have detrimental impacts on all forms of stream biota [63].

There were no statistically significant relationships between nutrient, turbidity, and TSS levels and the Time x Location interaction (P > 0.1 for all analyses; n = 275, 362, and 315 for each analysis at Pine, Bogard, and Bailey Creeks, respectively). This is in contrast to previous studies in which nutrient concentrations and sediment levels increased substantially following timber harvest activities [30,32,33,45,64,65]. These studies however involved the clear-cutting of large portions of the watershed. Our finding are consistent with studies on partial harvesting adjacent to streams and rivers with the implementation of BMPs, in which there are limited to no effects on nutrient concentrations or sediment levels [31-33,40-42,66].

The evaluation of NO_3_-N, NH_4_-N, and PO_4_-P across all stations and years at Pine, Bogard, and Bailey Creeks revealed extremely clean water conditions both before and after treatment implementation (Table 1). More than 80 % of all samples analyzed for NO_3_-N, NH_4_-N, and PO_4_-P were below the detection limit, with the exception of PO_4_-P in Bogard Creek, for which 14 % of samples were below the detection limit. The concentrations of nutrients measured across all stations from 2003 through 2010 are consistently below regulatory standards and similar to levels measured in unimpaired streams in the United States [67-69]. Such low levels of nutrients are characteristic of western montane forests [67,70-72].

**Table 1:** Results of nutrient analyses at Pine, Bogard, and Bailey Creeks.

**Creek**	**Nutrient**	**No. samples collected**	**No. samples < DL** ^a^	**% Samples < DL**	**Mean of all samples**	**Mean of samples > DL**
**Pine**	NO_3_-N	275	232	84	0.007	0.033
	NH_4_-N	275	273	99	0.026	0.137
	PO_4_-P	275	249	91	0.008	0.035
**Bogard**	NO_3_-N	362	298	82	0.007	0.028
	NH_4_-N	364	364	100	0.025	--
	PO_4_-P	362	49	14	0.037	0.042
**Bailey**	NO_3_-N	315	272	86	0.005	0.021
	NH_4_-N	316	293	93	0.030	0.091
	PO_4_-P	315	311	99	0.006	0.041

Summary of nitrate as N (NO_3_-N), ammonium as N (NH_4_-N), and phosphate as P (PO_4_-P) data collected for the PC1 to PC4 reach at Pine Creek (2003-2010), the BO1 to BO5 reach at Bogard Creek (2003-2010), and the BR1 to BR6 reach at Bailey Creek (2003-2004, 2006-2010).

^a^ DL = detection limit; The detection limit for NO_3_-N is 0.005 mg L^-1^, for NH_4_-N is 0.05 mg L^-1^, and for PO_4_-P is 0.01 mg L^-1^.

Tables 2-7 report the TSS, turbidity, discharge, temperature, and nutrient levels observed on Pine, Bogard, and Bailey Creeks immediately upstream and downstream of treatment areas. Turbidity and TSS levels did rise and fall across years, however these changes were not associated with treatment implementation (Turbidity and TSS levels are illustrated in Figures S1, S2, S3, S4, S5 and S6) (Tables 2, 4, and 6). For example, the highest turbidity levels over the entire study period at Bailey Creek were observed in 2004, before conifer removal took place (Table 6). High temporal variability in sediments has been detected in previous studies on the effects of timber harvesting on water quality [27,45,66]. Such variation is a result of the fact that all streams naturally gain and lose sediment along their length, and this gain-loss will vary from year to year [73,74]. A functioning stream will ultimately achieve a balance between sediment gain and loss. Lastly, although peaks in turbidity and TSS do occur, such as at station BO5 in 2004 (Table 4), it should be noted that these relatively elevated turbidity and TSS levels consistently return to normal levels in stations located immediately downstream and do not reappear in following years. This indicates that the peaks in turbidity and TSS levels, whatever their cause, are limited both spatially and temporally. 

**Table 2:** Mean and maximum annual values of total suspended solids (TSS), turbidity, discharge and stream temperature measured at Pine Creek.

					**TSS**		**Turbidity**		**Discharge**			**Daily Water Temp**	
			**% Average annual**		**(mg L^-1^)**		**(ntu)**		**(m^3^ sec^-1^)**			**(°C)**	
**Treatment**	**Year**	**n=**	**precipitation** ^a^	**Station** ^b^	**Mean**	**Max**	**Mean**	**Max**	**Min**	**Mean**	**Max**	**Mean** ^c^	**Max** ^d^
Pre	2003	9	118	PC4	5.9	8.0	0.42	0.80	5	19	41	11	14
				PC3	5.1	8.6	0.37	0.58	7	12	15	11	14
				PC1	4.2	6.9	0.29	0.41	0.5	2	3	12	14
Post Phase 1	2004	10	98	PC4	3.4	7.1	0.57	1.48	3	5	10	10	13
				PC3	4.2	9.4	0.93	2.07	5	7	10	11	13
				PC1	4.1	9.4	0.87	1.77	0.2	2	2	12	15
Post Phase 1	2005	12	110	PC4	2.4	9.4	0.29	1.03	5	8	14	10	14
				PC3	4.1	11.2	0.49	2.23	7	10	15	11	14
				PC1	3.9	8.2	0.58	1.46	0.03	0.5	2	12	15
Post Phase 2	2006	9	170	PC4	2.0	4.1	0.13	0.35	10	17	44	10	12
				PC3	2.1	4.1	0.26	0.64	12	19	32	10	12
				PC1	2.9	8.8	0.51	1.58	0.07	2	3	11	14
Post Phase 2	2007	9	73	PC4	2.4	10.0	0.28	1.08	2	3	10	11	16
				PC3	2.1	4.7	0.64	3.07	2	3	5	12	16
				PC1	2.7	4.1	0.30	0.53	0.02	0.03	0.03	11	17
Post Phase 3	2008	8	76	PC4	3.3	11.8	0.81	2.42	2	3	5	13	18
				PC3	2.5	4.1	0.45	0.93	2	2	5	13	18
				PC1	2.1	4.1	0.79	1.69	0.02	0.03	0.05	10	15
Post Phase 3	2009	9	99	PC4	3.8	5.3	0.32	0.76	3	5	7	12	16
				PC3	3.9	8.2	0.38	0.75	3	5	10	13	17
				PC1	4.7	8.2	0.55	3.17	0.03	0.07	0.12	12	17
Post Phase 3	2010	7	102	PC4	3.5	5.9	0.57	1.58	5	8	12	11	15
				PC3	4.3	8.2	0.91	2.83	8	10	17	12	15
				PC1	2.8	8.2	0.80	2.94	0.07	0.2	0.7	12	16

Data was collected each year for locations immediately upstream (PC4) and downstream (PC3, PC1) of the aspen stands adjacent to Pine Creek treated during the Pine-Bogard Project.

^a^ Average annual precipitation is 630 mm per year [48].

^b^ PC4 = upstream of all treatment areas; PC3 = downstream of Phase 1 treatment area; PC1 = downstream of Phase 2 and 3 treatment areas.

^c^ The standard error of mean daily water temperature measurements was always less than 1.3 °C.

^d^ The annual average of the daily maximum stream temperatures.

**Table 3:** Mean annual nutrient concentrations measured at Pine Creek.

				**NO_3_-N** ^b^		**NH_4_-N** ^c^		**PO_4_-P** ^d^		**SO_4_-S** ^d^		**K** ^e^
**Treatment**	**Year**	**n=**	**Station** ^a^	**mg L^-1^**	**%<DL** ^f^	**mg L^-1^**	**%<DL**	**mg L^-1^**	**%<DL**	**mg L^-1^**	**%<DL**	**mg L^-1^**
Pre	2003	9	PC4	0.0025 (0)	100	0.025 (0)	100	0.005 (0)	100	0.01 (0)	67	1.39 (0.08)
			PC3	0.003 (0.001)	88	0.025 (0)	100	0.01 (0)	75	0.01 (0)	25	1.38 (0.10)
			PC1	0.0025 (0)	100	0.025 (0)	100	0.005 (0)	100	0.01 (0)	44	1.42 (0.10)
Post Phase 1	2004	10	PC4	0.003 (0)	90	0.025 (0)	100	0.005 (0)	100	0.10 (0.01)	0	1.28 (0.05)
			PC3	0.0025 (0)	100	0.025 (0)	100	0.01 (0)	90	0.09 (0.01)	0	1.32 (0.08)
			PC1	0.0025 (0)	100	0.025 (0)	100	0.005 (0)	100	0.08 (0.02)	30	1.36 (0.07)
Post Phase 1	2005	12	PC4	0.003 (0.001)	92	0.025 (0)	100	0.01 (0)	67	0.06 (0)	0	1.51 (0.07)
			PC3	0.003 (0.001)	83	0.025 (0)	100	0.01 (0)	67	0.06 (0.01)	0	1.60 (0.14)
			PC1	0.004 (0.001)	90	0.025 (0)	100	0.01 (0)	80	0.04 (0.01)	20	1.52 (0.08)
Post Phase 2	2006	9	PC4	0.019 (0.016)	89	0.025 (0)	100	0.005 (0)	100	0.11 (0.02)	22	1.30 (0.05)
			PC3	0.0025 (0)	100	0.025 (0)	100	0.05 (0.04)	89	0.15 (0.02)	11	1.31 (0.05)
			PC1	0.0025 (0)	100	0.025 (0)	100	0.005 (0)	100	0.11 (0.02)	22	1.29 (0.06)
Post Phase 2	2007	9	PC4	0.0025 (0)	100	0.04 (0.01)	89	0.005 (0)	100	0.08 (0.02)	33	1.50 (0.10)
			PC3	0.0025 (0)	100	0.025 (0)	100	0.005 (0)	100	0.08 (0.01)	11	1.50 (0.09)
			PC1	0.0025 (0)	100	0.025 (0)	100	0.005 (0)	100	0.07 (0.02)	20	1.86 (0.41)
Post Phase 3	2008	8	PC4	0.049 (0.032)	25	0.025 (0)	100	0.01 (0)	88	0.09 (0.01)	13	1.46 (0.08)
			PC3	0.032 (0.013)	38	0.025 (0)	100	0.01 (0)	75	0.07 (0.02)	38	1.41 (0.07)
			PC1	0.016 (0.011)	50	0.025 (0)	100	0.02 (0.01)	75	0.27 (0.13)	25	1.31 (0.09)
Post Phase 3	2009	9	PC4	0.003 (0)	89	0.025 (0)	100	0.01 (0)	89	0.09 (0.01)	0	1.32 (0.05)
			PC3	0.0025 (0)	100	0.025 (0)	100	0.005 (0)	100	0.09 (0.01)	0	1.32 (0.04)
			PC1	0.003 (0.001)	86	0.025 (0)	100	0.005 (0)	100	0.07 (0.02)	29	1.33 (0.12)
Post Phase 3	2010	7	PC4	0.004 (0.001)	71	0.025 (0)	100	0.01 (0.01)	86	0.10 (0.01)	0	1.29 (0.11)
			PC3	0.008 (0.002)	43	0.04 (0.01)	86	0.01 (0)	86	0.09 (0.01)	0	1.30 (0.09)
			PC1	0.006 (0.002)	67	0.025 (0)	100	0.005 (0)	100	0.04 (0.01)	33	1.46 (0.20)

Nitrate as N (NO_3_-N), ammonium as N (NH_4_-N), phosphate as P (PO_4_-P), sulfate as S (SO_4_-S), and potassium (K) data was collected at locations immediately upstream (PC4) and downstream (PC3, PC1) of the aspen stands adjacent to Pine Creek treated during the Pine-Bogard Project.

Values in parenthesis are the standard error of the mean.

^a^ PC4 = upstream of all treatment areas; PC3 = downstream of Phase 1 treatment area; PC1 = downstream of Phase 2 and 3 treatment areas.

^b^ Concentrations below NO_3_-N detection level were set to 0.0025 mg L^-1^ which is one-half the detection level for this analysis (0.005 mg L^-1^).

^c^ Concentrations below NH_4_-N detection level were set to 0.025 mg L^-1^ which is one-half the detection level for this analysis (0.05 mg L^-1^).

^d^ Concentrations below PO_4_-P and SO_4_-S detection level were set to 0.005 mg L^-1^ which is one-half the detection level for this analysis (0.01 mg L^-1^).

^e^ There were no K concentrations below the detection level of 0.05 mg L^-1^.

^f^ Percent of samples below the detection limit.

**Table 4:** Mean and maximum annual values of total suspended solids (TSS), turbidity, discharge and stream temperature measured at Bogard Creek.

					**TSS**		**Turbidity**		**Discharge**			**Daily Water Temp**	
			**% Average annual**		**mg L^-1^**		**ntu**		**(m^3^ sec^-1^)**			**(°C)**	
**Treatment**	**Year**	**n=**	**precipitation** ^a^	**Station** ^b^	**Mean**	**Max**	**Mean**	**Max**	**Min**	**Mean**	**Max**	**Mean** ^c^	**Max** ^d^
Pre	2003	9	118	BO5	6.9	17.1	0.76	1.57	0.8	1.4	2.0	9	15
				BO3	7.8	12.6	1.09	2.78	0.8	1.5	2.7	10	16
				BO1	5.8	11.4	1.04	2.02	0.5	1.0	1.7	10	15
Post Phase 1	2004	10	98	BO5	16.3	35.3	2.71	5.23	0.8	1.5	2.4	9	14
				BO3	7.6	20.6	1.27	3.22	0.7	1.2	1.7	10	15
				BO1	7.4	15.3	2.39	5.51	0.3	0.8	1.2	10^e^	15^e^
Post Phase 1	2005	12	110	BO5	4.5	14.7	0.66	2.44	0.8	1.2	1.7	8	14
				BO3	3.6	10.0	0.82	2.44	0.5	1.0	1.5	9	15
				BO1	3.2	6.5	0.78	3.42	0.3	0.7	1.5	10	14
Post Phase 2	2006	10	170	BO5	3.3	8.8	0.38	0.88	3.1	4.9	7.1	8	10
				BO3	3.1	5.3	0.37	0.68	3.2	4.8	6.3	8	12
				BO1	2.7	4.1	0.37	0.76	2.7	4.2	6.8	8	12
Post Phase 2	2007	9	73	BO5	10.3	24.7	2.26	4.01	0.5	0.7	0.8	10	13
				BO3	5.3	18.2	1.25	4.89	0.3	0.5	0.8	11	17
				BO1	7.6	17.1	2.06	6.41	0.2	0.3	0.5	11	15
Post Phase 3	2008	8	76	BO5	5.3	15.9	2.01	5.71	0.2	0.2	0.3	12	18
				BO3	5.1	9.4	1.47	4.82	0.2	0.2	0.3	13	19
				BO1	7.6	14.7	2.11	3.97	0.05	0.2	0.3	13	20
Post Phase 3	2009	9	99	BO5	8.0	17.7	2.55	5.57	0.2	0.2	0.3	12	17
				BO3	7.6	13.5	1.45	3.77	0.05	0.3	1.0	13	19
				BO1	8.0	22.9	1.22	2.73	0.2	0.2	0.5	13	19
Post Phase 3	2010	7	102	BO5	5.2	10.0	1.26	2.99	0.2	1.0	2.2	11	16
				BO3	6.5	11.2	2.08	3.04	0.2	0.5	1.0	12	17
				BO1	6.2	8.8	2.00	4.27	0.05	0.5	1.4	13	17

Data was collected each year for locations immediately upstream (BO5) and downstream (BO3, BO1) of the aspen stands adjacent to Bogard Creek treated during the Pine-Bogard Project.

^a^ Average annual precipitation is 630 mm per year [48].

^b^ BO5 = upstream of treatment areas; BO3 = downstream of Phase 1 treatment area; BO1 = downstream of Phase 2 and 3 treatment areas.

^c^ The standard error of mean daily water temperature measurements was always less than 1.0 °C.

^d^ The annual average of the daily maximum stream temperatures.

^e^ 2004 stream temperature values at station BO1 are from station BO2 because logger at station BO1 was broken.

**Table 5:** Mean annual nutrient concentrations measured at Bogard Creek.

				**NO_3_-N** ^b^		**NH_4_-N** ^c^		**PO_4_-P** ^d^		**SO_4_-S** ^d^		**K** ^e^
**Treatment**	**Year**	**n=**	**Station** ^a^	**mg L^-1^**	**%<DL** ^f^	**mg L^-1^**	**%<DL**	**mg L^-1^**	**%<DL**	**mg L^-1^**	**%<DL**	**mg L^-1^**
Pre	2003	9	BO5	0.0025 (0)	100	0.025 (0)	100	0.03 (0.01)	13	0.02 (0)	13	2.16 (0.10)
			BO3	0.0025 (0)	100	0.025 (0)	100	0.03 (0.01)	11	0.03 (0)	0	2.25 (0.07)
			BO1	0.0025 (0)	100	0.025 (0)	100	0.05 (0.02)	11	0.03 (0)	0	2.29 (0.20)
Post Phase 1	2004	10	BO5	0.003 (0.001)	80	0.025 (0)	100	0.01 (0)	40	0.18 (0.01)	0	1.99 (0.06)
			BO3	0.0025 (0)	100	0.025 (0)	100	0.01 (0)	44	0.18 (0.01)	0	1.91 (0.04)
			BO1	0.0025 (0)	100	0.025 (0)	100	0.01 (0)	44	0.16 (0.01)	0	1.95 (0.05)
Post Phase 1	2005	12	BO5	0.0025 (0)	100	0.025 (0)	100	0.04 (0)	0	0.13 (0.01)	0	2.27 (0.04)
			BO3	0.004 (0.001)	83	0.025 (0)	100	0.04 (0)	0	0.12 (0.01)	0	2.16 (0.08)
			BO1	0.003 (0)	92	0.025 (0)	100	0.04 (0)	0	0.13 (0.01)	0	2.12 (0.06)
Post Phase 2	2006	10	BO5	0.0025 (0)	100	0.025 (0)	100	0.04 (0.01)	0	0.23 (0.02)	0	2.06 (0.04)
			BO3	0.0025 (0)	100	0.025 (0)	100	0.05 (0.01)	0	0.24 (0.02)	0	2.05 (0.05)
			BO1	0.0025 (0)	100	0.025 (0)	100	0.04 (0.01)	0	0.25 (0.01)	0	2.03 (0.04)
Post Phase 2	2007	9	BO5	0.004 (0.001)	78	0.025 (0)	100	0.04 (0)	0	0.20 (0.01)	0	2.44 (0.31)
			BO3	0.005 (0.003)	89	0.025 (0)	100	0.04 (0.01)	0	0.19 (0.01)	0	2.51 (0.26)
			BO1	0.0025 (0)	100	0.025 (0)	100	0.03 (0)	0	0.20 (0.02)	0	2.34 (0.23)
Post Phase 3	2008	8	BO5	0.007 (0.003)	75	0.025 (0)	100	0.06 (0.01)	0	0.21 (0.01)	0	2.01 (0.04)
			BO3	0.013 (0.006)	63	0.025 (0)	100	0.07 (0.01)	0	0.23 (0.02)	0	2.00 (0.11)
			BO1	0.005 (0.002)	75	0.025 (0)	100	0.04 (0.01)	13	0.20 (0.01)	0	1.99 (0.11)
Post Phase 3	2009	9	BO5	0.019 (0.007)	44	0.025 (0)	100	0.03 (0.01)	22	0.17 (0.01)	0	1.86 (0.09)
			BO3	0.016 (0.006)	56	0.025 (0)	100	0.01 (0)	56	0.18 (0.01)	0	1.87 (0.12)
			BO1	0.021 (0.014)	67	0.025 (0)	100	0.01 (0)	44	0.16 (0.02)	11	1.82 (0.13)
Post Phase 3	2010	7	BO5	0.005 (0.001)	57	0.025 (0)	100	0.04 (0.01)	14	0.17 (0.02)	0	1.98 (0.10)
			BO3	0.006 (0.002)	57	0.025 (0)	100	0.05 (0.01)	14	0.19 (0.01)	0	1.90 (0.13)
			BO1	0.004 (0.001)	71	0.025 (0)	100	0.04 (0.01)	14	0.16 (0.01)	0	1.87 (0.14)

Nitrate as N (NO_3_-N), ammonium as N (NH_4_-N), phosphate as P (PO_4_-P), sulfate as S (SO_4_-S), and potassium (K) data was collected at locations immediately upstream (BO5) and downstream (BO3, BO1) of the aspen stands adjacent to Bogard Creek treated during the Pine-Bogard Project.

Values in parenthesis are the standard error of the mean.

^a^ BO5 = upstream of treatment areas; BO3 = downstream of Phase 1 treatment area; BO1 = downstream of Phase 2 and 3 treatment areas.

^b^ Concentrations below NO_3_-N detection level were set to 0.0025 mg L^-1^ which is one-half the detection level for this analysis (0.005 mg L^-1^).

^c^ Concentrations below NH_4_-N detection level were set to 0.025 mg L^-1^ which is one-half the detection level for this analysis (0.05 mg L^-1^).

^d^ Concentrations below PO_4_-P and SO_4_-S detection level were set to 0.005 mg L^-1^ which is one-half the detection level for this analysis (0.01 mg L^-1^).

^e^ There were no K concentrations below the detection level of 0.05 mg L^-1^.

^f^ Percent of samples below the detection limit.

**Table 6:** Mean and maximum annual values of total suspended solids (TSS), turbidity, discharge and stream temperature measured at Bailey Creek.

					**TSS**		**Turbidity**		**Discharge**			**Daily Water Temp**	
			**% Average annual**		**mg L^-1^**		**ntu**		**(m^3^ sec^-1^)**			**(°C)**	
**Treatment**	**Year**	**n=**	**precipitation** ^a^	**Station** ^b^	**Mean**	**Max**	**Mean**	**Max**	**Max**	**Mean**	**Min**	**Mean** ^c^	**Max** ^d^
Pre	2003	6	107	BR6	4.3	6.9	0.41	0.67	59	29	8	8	10
				BR1	5.3	10.0	0.60	0.99	92	46	17	9	12
Pre	2004	8	93	BR6	2.5	7.7	1.02	3.10	58	24	5	8	11
				BR1	3.0	6.5	1.22	3.88	71	31	8	9	12
Pre	2006	7	117	BR6	2.7	6.5	0.21	0.62	54	24	7	7	10
September				BR1	3.2	5.3	0.18	0.28	65	29	7	8	11
Post	2007	8	51	BR6	1.6	3.5	0.25	0.53	19	8	3	10	13
				BR1	1.5	3.5	0.33	0.79	20	8	3	11	15
Post	2008	9	55	BR6	3.3	14.1	0.37	1.27	42	12	3	10	13
				BR1	1.7	5.9	0.52	1.08	46	15	3	11	15
Post	2009	8	69	BR6	3.7	5.3	0.56	1.95	34	17	3	9	12
				BR1	4.5	7.1	0.43	0.71	41	22	10	10	14
Post	2010	7	78	BR6	5.3	13.5	0.82	3.01	78	37	7	7	10
				BR1	6.5	8.8	0.83	1.54	134	53	19	8	11

Data was collected each year for locations immediately upstream (BR6) and downstream (BR1) of the aspen stands adjacent to Bailey Creek treated during the Bailey Project.

^a^ Average annual precipitation is 1,590 mm per year [48].

^b^ BR6 = upstream of treatment areas; BR1 = downstream of treatment areas.

^c^ The standard error of mean daily water temperature measurements was always less than 1.9 °C.

^d^ The annual average of the daily maximum stream temperatures.

**Table 7:** Mean annual nutrient concentrations measured at Bailey Creek.

				**NO_3_-N** ^b^		**NH_4_-N** ^c^		**PO_4_-P** ^d^		**SO_4_-S** ^e^	**K** ^f^
**Treatment**	**Year**	**n=**	**Station** ^a^	**mg L^-1^**	**%<DL** ^g^	**mg L^-1^**	**%<DL**	**mg L^-1^**	**%<DL**	**mg L^-1^**	**mg L^-1^**
Pre	2003	6	BR6	0.004 (0.001)	67	0.06 (0.02)	50	0.005 (0)	100	3.65 (0.52)	0.91 (0.22)
			BR1	0.0025 (0)	100	0.06 (0.02)	60	0.005 (0)	100	3.19 (0.45)	0.74 (0.07)
Pre	2004	8	BR6	0.003 (0.001)	88	0.03 (0.01)	88	0.005 (0)	100	4.46 (0.59)	0.69 (0.07)
			BR1	0.004 (0.001)	88	0.025 (0)	100	0.005 (0)	100	4.22 (0.51)	0.70 (0.07)
Pre	2006	7	BR6	0.009 (0.007)	86	0.025 (0)	100	0.005 (0)	100	3.99 (0.55)	0.75 (0.06)
September			BR1	0.0025 (0)	100	0.025 (0)	100	0.005 (0)	100	3.88 (0.50)	0.77 (0.07)
Post	2007	8	BR6	0.035 (0.032)	88	0.025 (0)	100	0.005 (0)	100	5.72 (0.54)	0.79 (0.06)
			BR1	0.0025 (0)	100	0.04 (0.01)	75	0.005 (0)	100	5.35 (0.42)	0.86 (0.07)
Post	2008	9	BR6	0.0025 (0)	100	0.025 (0)	100	0.01 (0.01)	78	5.48 (0.65)	0.79 (0.05)
			BR1	0.014 (0.010)	67	0.025 (0)	100	0.005 (0)	100	5.04 (0.53)	0.84 (0.06)
Post	2009	8	BR6	0.0025 (0)	100	0.025 (0)	100	0.005 (0)	100	4.85 (0.56)	0.74 (0.06)
			BR1	0.0025 (0)	100	0.025 (0)	100	0.005 (0)	100	4.55 (0.50)	0.77 (0.07)
Post	2010	7	BR6	0.007 (0.003)	57	0.025 (0)	100	0.005 (0)	100	3.18 (0.57)	0.70 (0.07)
			BR1	0.005 (0.001)	33	0.025 (0)	100	0.005 (0)	100	2.89 (0.51)	0.75 (0.08)

Nitrate as N (NO_3_-N), ammonium as N (NH_4_-N), phosphate as P (PO_4_-P), sulfate as S (SO_4_-S), and potassium (K) data was collected at locations immediately upstream (BR1) and downstream (BR6) of the aspen stands adjacent to Bailey Creek treated during the Bailey Project.

Values in parenthesis are the standard error of the mean.

^a^ BR6 = upstream of treatment areas; BR1 = downstream of treatment areas.

^b^ Concentrations below NO_3_-N detection level were set to 0.0025 mg L^-1^, which is one-half the detection level for this analysis (0.005 mg L^-1^).

^c^ Concentrations below NH_4_-N detection level were set to 0.025 mg L^-1^, which is one-half the detection level for this analysis (0.05 mg L^-1^).

^d^ Concentrations below PO_4_-P detection level were set to 0.005 mg L^-1^, which is one-half the detection level for this analysis (0.01 mg L^-1^).

^e^ There were no SO_4_-S concentrations below the SO_4_-S detection level of 0.01 mg L^-1^.

^f^ There were no K concentrations below the K detection level of 0.05 mg L^-1^.

^g^ Percent of samples below the detection limit.

Nutrient concentrations exhibited little variation between years and between stations (Tables 3, 5, and 7; Figures S7, S8, S9, S10, S11, S12, S13, S14, and S15). With the exception of PO_4_-P in Bogard Creek, the spatial and temporal consistency observed in nutrient concentrations is likely the result of the overall nutrient limitation of the creeks. The lack of variation in PO_4_-P levels between stations and years at Bogard Creek (Table 5; Figure S14) implies that the elevated PO_4_-P concentrations are a natural characteristic of Bogard Creek rather than the result of a treatment effect. As described in Section 2.1, Bogard Creek flow consists of a high fraction of sub-surface inputs, which can derive substantial quantities of P from soils and bedrock [67,75,76]. However, despite relatively elevated PO_4_-P concentrations, the low levels of N in Bogard Creek likely limit the potential of P to cause eutrophication [77]. 

### 3.2: Solar Radiation and Canopy Cover

Vegetative canopy cover is a critical factor in aquatic ecosystems because it blocks solar radiation reaching stream surfaces and thus moderates water temperature [35] and influences in-stream primary production [78,79]. Additionally, vegetative canopy cover also serves as an input of nutrients and organic matter to stream systems and provides physical habitat for stream biota [34,80]. 

There were no statistically significant changes in canopy cover along the treatment reaches of Pine Creek (PC4 to PC1) or Bogard Creek (BO5 to BO1)in response to Pine-Bogard Phase 2 conifer removal (P > 0.1; n = 74 and 76 at Pine and Bogard Creeks, respectively). Mean canopy cover at Pine Creek was 66 % before treatment and 64 % after treatment. Mean canopy cover at Bogard Creek was 64 % before treatment and 62 % after treatment. Correspondingly, there were no significant changes in the potential fraction of solar radiation arriving at each creek (P > 0.1; n = 74 and 76 at Pine and Bogard Creeks, respectively). Mean solar radiation values for the months of May through September at Pine Creek were 31, 33, 32, 26, and 21 % before treatment and 33, 33, 33, 31, and 25 % after treatment. Mean solar radiation values for the months of May through September at Bogard Creek were 36, 38, 37, 30, and 22 % before treatment and 40, 44, 42, 31, 20 % after treatment. The lack of significant change is not surprising, given that Phase 2 conifer removal occurred primarily outside of areas immediately adjacent to Pine and Bogard Creeks (Figure 2). 

**Figure 4:**
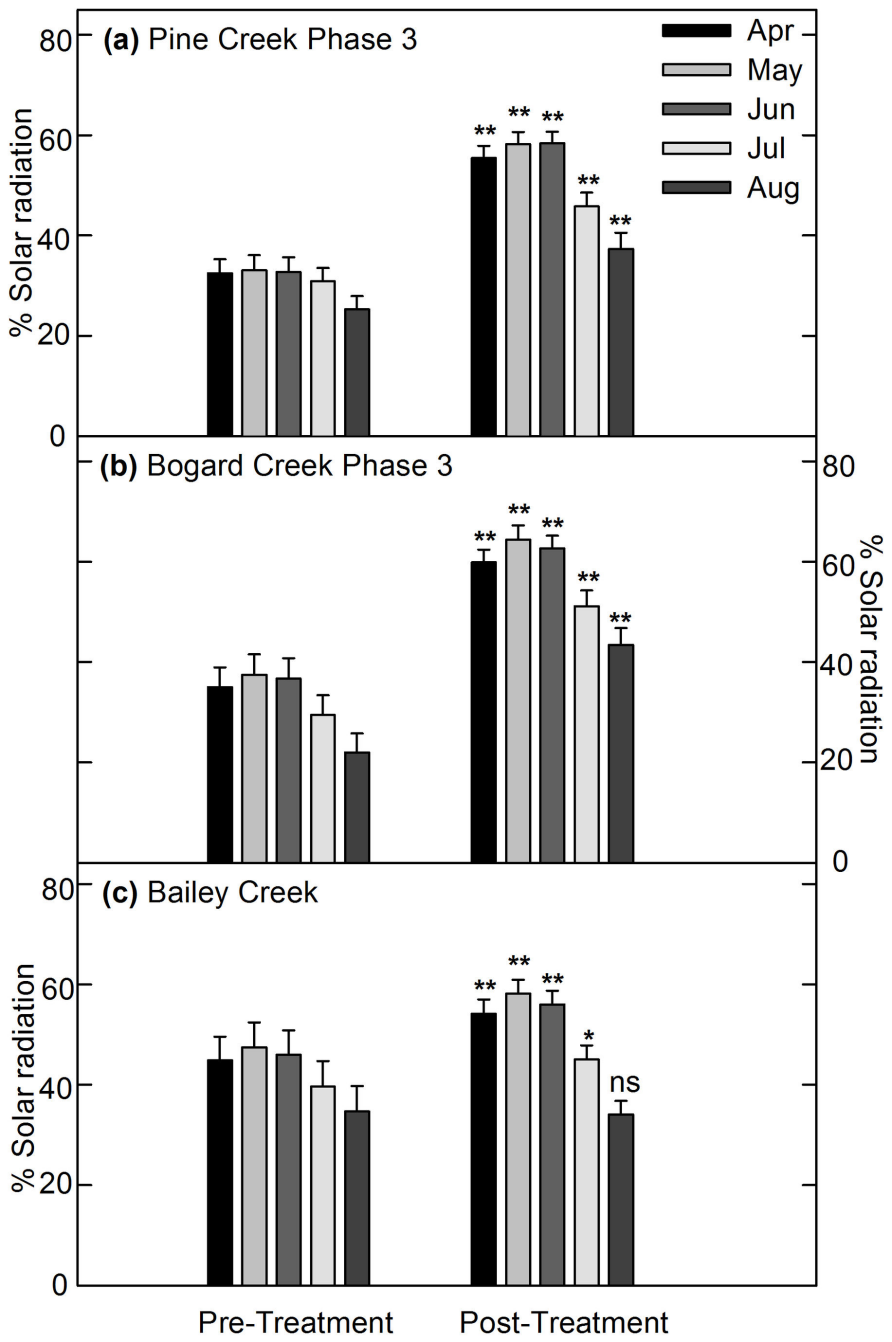
Mean and standard error of the potential fraction of solar radiation arriving at Pine, Bogard, and Bailey Creeks. (a) Pine Creek between stations PC4 and PC1 before (2005) and after (2008) January 2008 Phase 3 conifer removal, (b) Bogard Creek between stations BO5 and BO1 before (2005) and after (2008) January 2008 Phase 3 conifer removal, and (c) Bailey Creek between stations BR6 and BR1 before (2003) and after (2007) September 2006 conifer removal. * indicates significantly different at P<0.05. ** indicates significantly different at P<0.005.

There were significant decreases in canopy cover along the treatment reaches of Pine Creek (PC1 to PC4) and Bogard Creek (BO1 to BO5) in response to Pine-Bogard Phase 3 conifer removal (P < 0.002; n = 106 at both Pine and Bogard Creeks ). Canopy cover decreased from a mean of 64 to 55 % along Pine Creek and from a mean of 64 to 39 % along Bogard Creek. Correspondingly, there was a significant increase in the potential fraction of solar radiation arriving at each reach (P < 0.005; n = 75 and 81 at Pine and Bogard Creeks, respectively). Figures 4a and b report the mean solar radiation results before and after January 2008 conifer removal. The magnitude of the increase in the potential fraction of solar radiation arriving at Pine Creek from the months of May through September ranged from 12 % in September to 25 % in June and July. The magnitude of the increase in the potential fraction of solar radiation arriving at Bogard Creek from the months of May through September ranged from 21 % in September to 27 % in June and July. The significant decreases in canopy cover and increases in solar radiation were expected, as Phase 3 conifer removal was carried out directly adjacent to Pine and Bogard Creeks.

Canopy cover significantly decreased from a mean of 64 to 55 % along the treatment reach of Bailey Creek (BR1 to BR6) in response to conifer removal (P < 0.005; n = 82). Correspondingly, there was a significant increase in the potential fraction of solar radiation arriving at Bailey Creek following treatment for the months of May through August (P < 0.01; n = 82). Figure 4c reports the mean solar radiation results before and after the September 2006 conifer removal. The magnitude of the increase in the potential fraction of solar radiation ranged from 5 % in August to 11 % in June. The significant decreases in canopy cover and increases solar radiation were expected, as portions of Bailey Creek conifer removal were carried out directly adjacent to the stream.

### 3.3: Stream Temperature

**Figure 5:**
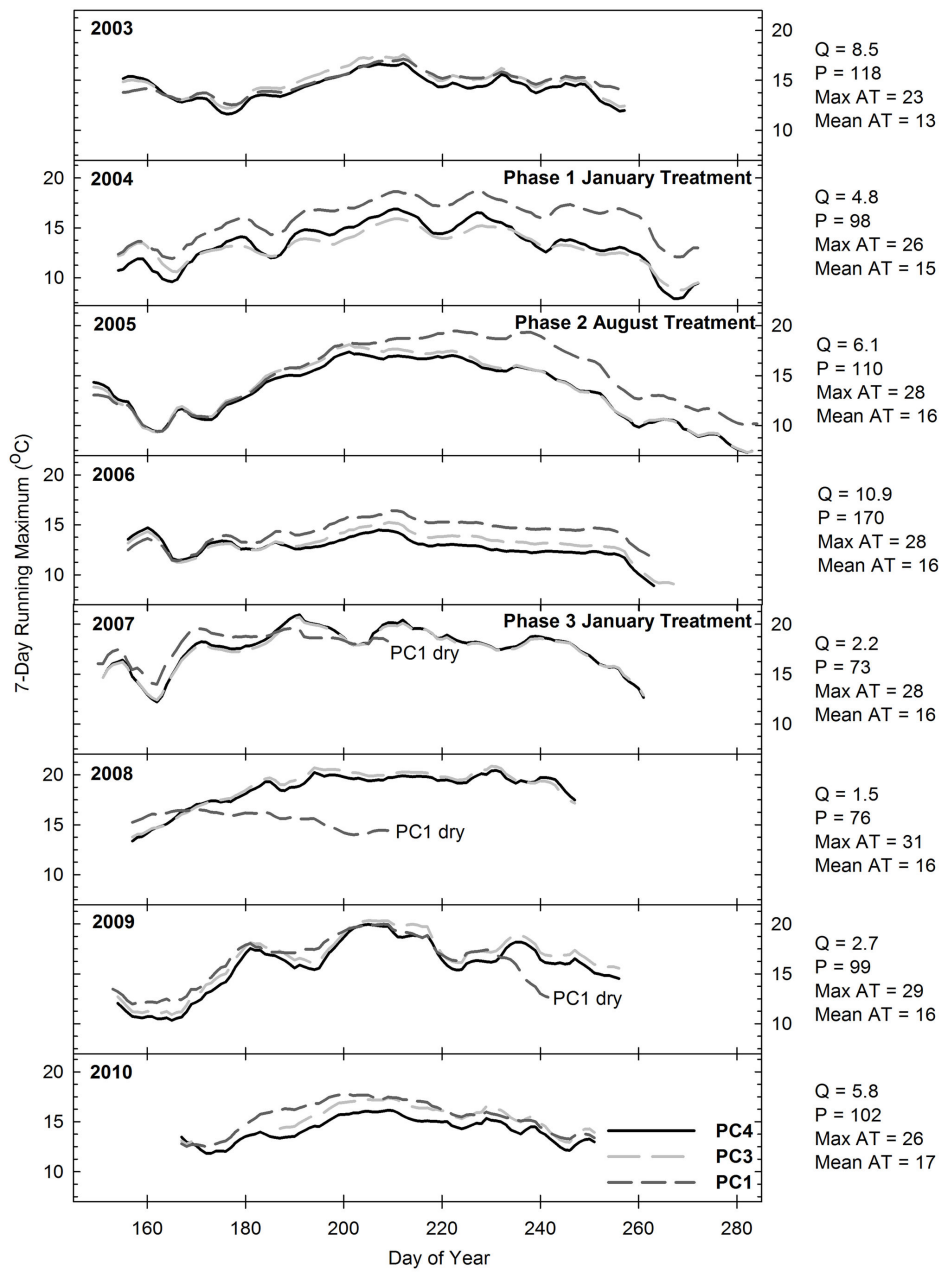
Seven day running average daily maximum water temperature (°C) on Pine Creek. Station PC4 was located immediately upstream of the areas treated during Phases 1-3. Station PC3 was located immediately downstream of the areas treated during Phase 1, and station PC1 was located immediately downstream of the areas treated during Phases 2 and 3. Q = mean discharge (m^3^ min^-1^) from Jun 15 – Aug 31, P = percent of mean annual precipitation, Max AT = mean of daily maximum air temperature (°C) from Jun 15 – Aug 31, AT = mean of average daily air temperature (°C) from Jun 15 – Aug 31.

**Figure 6:**
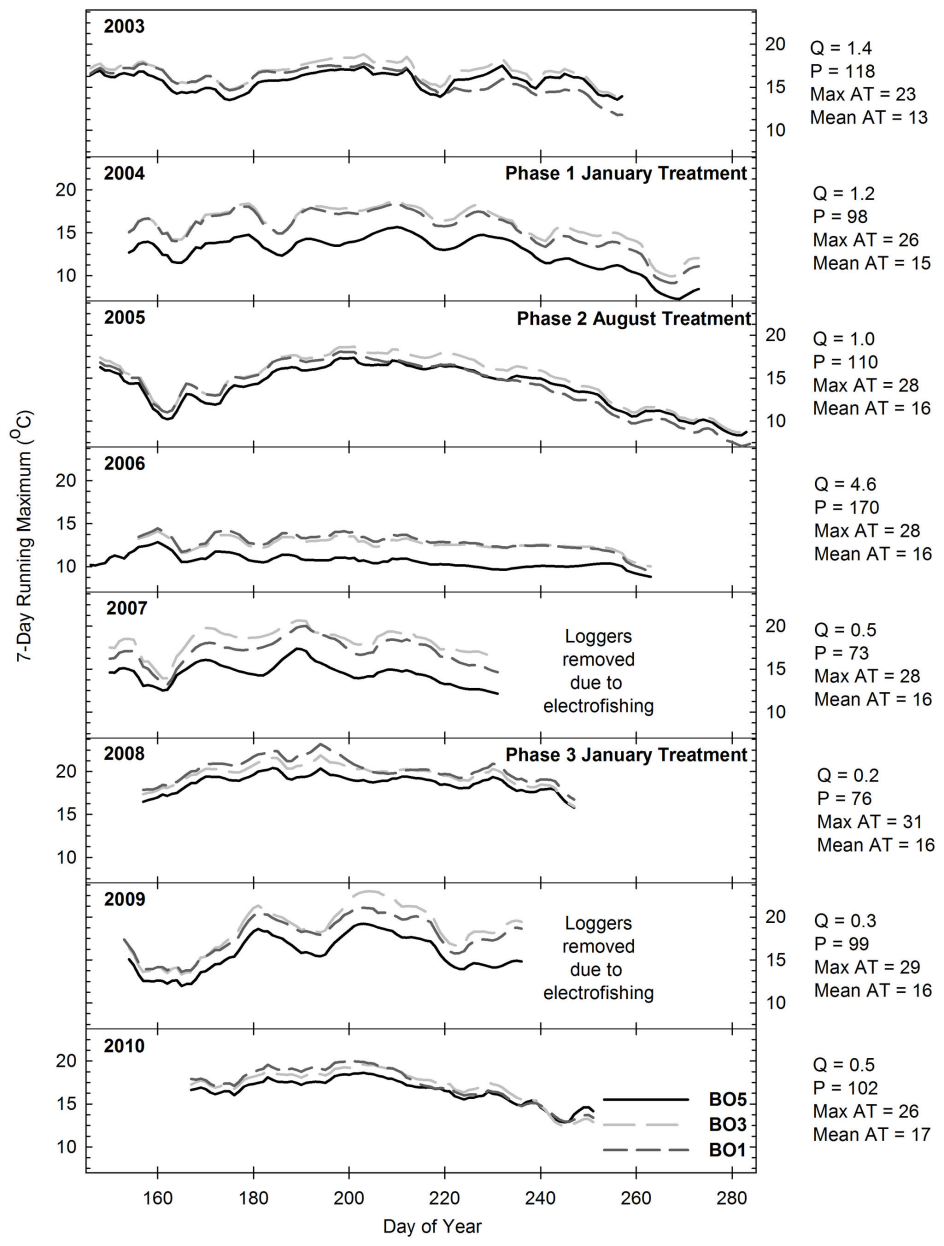
Seven day running average daily maximum water temperature (°C) on Bogard Creek. Station BO5 was located immediately upstream of the areas treated during Phases 1-3. Station BO3 was located immediately downstream of the areas treated during Phase 1, and station BO1 was located immediately downstream of the areas treated during Phases 2 and 3. Q = mean discharge (m^3^ min^-1^) from June 15 - August 31, P = percent of mean annual precipitation, Max AT = mean of daily maximum air temperature (°C) from June 15 - August 31, AT = mean of average daily air temperature (°C) from June 15 - August 31. Note: the temperature logger for station BO1 in 2004 was broken, so the 2004 figure shows data collected at station BO2.

**Figure 7:**
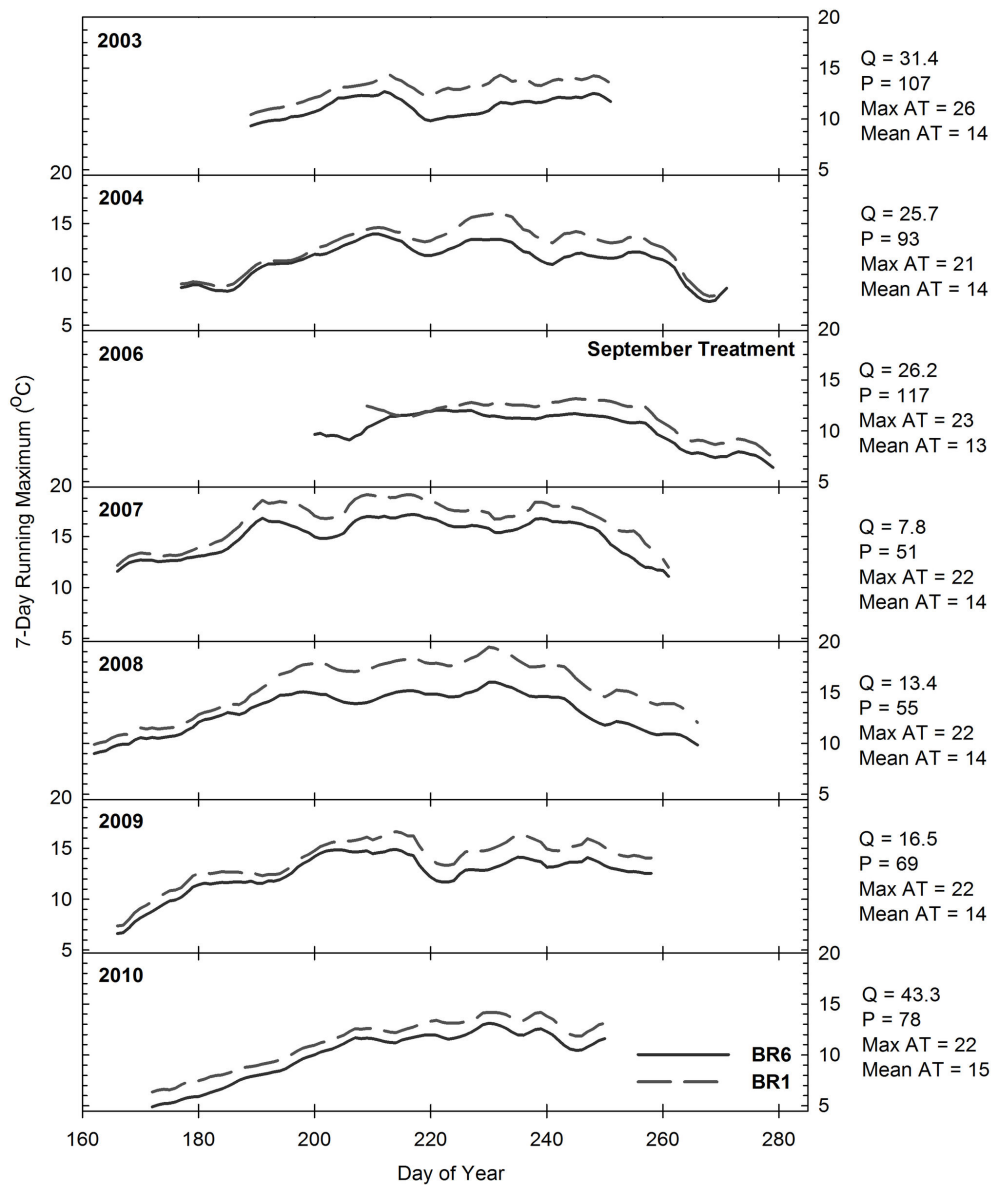
Seven day running average daily maximum water temperature (°C) on Bailey Creek. Stations BR6 and BR1 were located immediately upstream and downstream of the areas treated during the Bailey Project, respectively. Q = mean discharge (m^3^ min^-1^) from Jun 15 – Aug 31, P = percent of mean annual precipitation, Max AT = mean of daily maximum air temperature (°C) from Jun 15 – Aug 31, AT = mean of average daily air temperature (°C) from Jun 15 – Aug 31.

There was no statistically significant relationship between stream temperature and the Time x Location interaction in any of the creeks (P > 0.1 for all analyses; n = 276, 334, and 305 at Pine, Bogard, and Bailey Creeks, respectively). Figures 5-7 report the 7-day running average daily maximum water temperatures observed on the upstream and downstream stations of Pine, Bogard, and Bailey Creeks. Annual stream temperature patterns appear to be driven primarily by discharge, with higher temperatures occurring in years with lower flow. The data indicates a general pattern of increased temperature from upstream to downstream stations along each creek. Although the rate of increase in stream temperature from upstream to downstream stations varied annually, Figures 5-7 confirm that it was not associated with conifer removal treatments. For example, there was no change in the rate of increase from PC4 to PC1 or from BO5 to BO1 following Phase 3 conifer removal (Figures 5 and 6). Additionally, the rate of increase at Bailey Creek was similar both before (2003-2006) and after (2007) treatment (Figure 7). It then rose in 2008, but declined again in 2009 and 2010. The lack of correspondence between the rate of increase and treatment implementation implies that fluctuations in the primary drivers of stream temperature, including groundwater inputs, hyporheic flow, air temperature and discharge [35], are the source of variation in the rates of temperature change across years. 

A response in stream temperature to treatment implementation was not expected to Pine-Bogard Phase 1 and Phase 2 conifer removal, as these treatments occurred primarily outside of areas immediately adjacent to the creeks (Figure 2) and Phase 2 was found to have no significant impact on canopy cover or the potential fraction solar radiation reaching the creeks. However, the Pine-Bogard Phase 3 and Bailey treatments significantly decreased canopy cover and increased the potential fraction of solar radiation reaching the creeks, and therefore had the potential to increase downstream temperatures. There are several possible reasons for the lack of response. First, the decrease in canopy cover was minimal at Pine and Bailey Creeks (9 % and 7 % decrease, respectively). Previous studies with similar reductions in canopy cover also found no temperature response [36,66,81,82]. Second, there was still a substantial amount of canopy cover remaining at Pine Creek (55 %), Bogard Creek (39 %), and Bailey Creek (45 %) which likely provided sufficient shading to continue to moderate stream temperature. Third, at Bailey Creek, stream temperature change is likely buffered by the relatively high, cool flows that characterize the creek all season-long (Table 7). Fourth, as discussed below, the soil moisture tension data indicates that soil moisture increased as a result of vegetation removal, and this implies that there may have been an increase in water inputs to the streams that helped to buffer stream temperature from potential increases in solar radiation inputs [8,36,83]. Lastly, it is likely that the affected reach lengths at each creek were not long enough to allow for a water residence time that could result in increased temperatures. This may particularly be the case at Bogard Creek, where there was a 35 % decrease in canopy cover, and therefore an increase in temperature was expected. A shorter residence time lessens the potential for water passing through a reach to be influenced by solar radiation arriving at that reach. Chizinski et al. [81] and Hemstad et al. [82] also hypothesized that the relatively short reach lengths impacted by harvest activities adjacent to streams are one of the reasons that they found no adverse effects on aquatic ecosystem parameters.

For the purposes of aspen restoration, the scale of the timber harvest areas in this study would likely be among the largest that would be carried out should this method be applied throughout this region. This implies that the future application of these aspen restoration treatments would be unlikely to increase stream temperatures; however, projects that involve timber harvesting along longer stream reaches than those investigated in this study should monitor stream temperatures and take measures to limit potential impacts, such as leaving ample understory for shading [36,66]. In general, studies in which the clear-cutting of watersheds took place both with and without riparian buffers have shown significant increases in temperature [84,85]. In contrast, the results of our project are consistent with studies in which partial harvesting in areas adjacent to streams and rivers was carried out following BMPs, which prevented or significantly limited stream temperature changes [36,41,66,86,87]. 

Minimizing increases in stream temperature is a critical part of maintaining aquatic ecosystem health for stream biota. Increases in stream temperature have been found to alter macroinvertebrate abundance and community structure [43,88]. Additionally, high temperatures can substantially decrease cold-water fish populations [89]. Studies have found that the optimal temperatures for rainbow trout (*Oncorhynchus mykiss*), which is the native trout species in the region, range from 16 to 18 °C, and that the upper incipient lethal temperature for rainbow trout is approximately 25 °C [90]. Tables 2, 4, and 6 indicate mean stream temperatures did not exceed the optimal range during the course of this study, and Figures 5-7 indicate that maximum stream temperatures did not exceed the upper incipient lethal temperature. The highest temperatures observed at any of the sites throughout the course of this study occurred in Bogard Creek during the low-flow years from 2007 through 2009 (Figure 6). Despite these elevated temperatures, there was an abundance of trout in Bogard Creek during this time period, reflecting the suitability of these creeks to provide habitat for cold water fish even with timber harvesting activities [91]. 

### 3.4: Aquatic Macroinvertebrate Metrics

Table 8 reports key aquatic macroinvertebrate metrics calculated from collections made on Pine, Bogard, and Bailey Creeks. A high value of the percent of the macroinvertebrate community tolerant of pollution (% Tolerant) is an indicator of poor aquatic ecosystem health. Conversely, high values of (i) the percent of the macroinvertebrate community not tolerant of pollution (% Intolerant), (ii) of the Shannon Diversity Index, and (iii) of the number of families detected (No. Families) are indicators of good aquatic ecosystem health. There were no statistically significant relationships between these metrics and the Time x Location interaction (P > 0.1; n = 10 for the analysis of each parameter at each creek). 

**Table 8:** Aquatic macroinvertebrate metrics for Pine, Bogard, and Bailey Creek samples collected in June-July of 2003-2004, 2007-2008, and 2010.

		**Midstream station** ^b^					**Upstream station** ^c^				
**Creek**	**Metric** ^a^	**2003**	**2004**	**2007**	**2008**	**2010**	**2003**	**2004**	**2007**	**2008**	**2010**
**Pine**	No. Families	17	17	19	23	26	16	11	21	25	21
	Shannon D.I.	2.34	2.62	1.13	1.40	1.84	2.37	1.68	1.02	1.50	1.81
	% Tolerant	0.2	0	0	0	0.1	0	0	0	0	0
	% Intolerant	16	25	7	8	10	16	14	4	7	8
**Bogard**	No. Families	17	17	15	31	22	12	20	21	20	23
	Shannon D.I.	2.83	2.09	2.76	2.69	2.67	2.00	2.22	2.23	1.91	2.25
	% Tolerant	0	0	0	0	0	0	0	0	0	0
	% Intolerant	24	13	21	18	22	29	11	17	7	11
**Bailey**	No. Families	11	12	12	15	21	13	10	12	18	17
	Shannon D.I.	2.03	2.19	1.96	2.49	2.78	2.31	2.01	2.08	2.59	2.92
	% Tolerant	0	0	0	0	0	0	0	0	0	0
	% Intolerant	50	37	27	30	53	23	52	33	42	38

^a^ No. Families = number of families; Shannon D.I. = Shannon Diversity Index; % Tolerant = percent of the macroinvertebrate community tolerant of pollution; % Intolerant = percent of the macroinvertebrate community intolerant of pollution.

^b^ Midstream stations were PC3, BO3, and BR4 at Pine, Bogard, and Bailey Creeks, respectively.

^c^ Upstream stations were PC4, BO5, and BR6 at Pine, Bogard, and Bailey Creeks, respectively.

The results indicate healthy in-stream habitat conditions across all three creeks throughout the course of this study. The value of % Tolerant was zero in 93 % of the samples collected across all stations and years. The highest value of % Tolerant was 0.2 % measured in 2003 at midstream station PC3 prior to treatment implementation. At each creek, the Shannon Diversity Index, No. Families, and % Intolerant varied from year to year, but variation of similar magnitudes occurred at all stations (upstream and midstream), and the annual changes were not indicative of a decline in aquatic ecosystem health in response to conifer removal treatments. These results parallel the findings of the Kreutzweiser et al. [41] study, which found that most changes in macroinvertebrate community metrics before versus after partial timber harvesting adjacent to two watershed streams were similar in magnitude to the changes detected at the reference watershed and concluded that the timber harvest activities studied did not cause degradation of macroinvertebrate community structure and function. Similarly, studies by Chizinski et al. [81] and Gravelle et al. [39] found high annual variation in macroinvertebrate metrics but no treatment effect in response to partial timber harvesting or clear cutting activities. Strong temporal variability in macroinvertebrate metrics has been commonly observed in aquatic ecosystems, particularly in response to high annual variation in rainfall, which is characteristic of Mediterranean climates [92-94]. This implies that the variability detected in this study reflects site specific responses to annual climatic variation rather than the effects of conifer removal treatments. 

Although strong temporal variability can mask treatment effects [95,96], the apparent lack of response of macroinvertebrate metrics to conifer removal treatments is likely the result of a lack of treatment effect on key stream characteristics. Timber harvest activities affect stream macroinvertebrate communities through several mechanisms. Aquatic macroinvertebrate community structure and function has been found to be degraded by increases in stream temperature due to reductions in canopy cover, by nutrient enrichment due to soil disturbance, and by fine inorganic sediment loading following timber harvesting [34,97,98]. Previous studies in which temperatures, sediments, and algae increased following timber harvest activities found negative impacts on the stream macroinvertebrate communities [38,78,99]. In contrast, stream parameters showed little to no response to the timber harvest activities carried out in the studies by Chizinski et al. [81], Hemstad et al. [82], and Kreutzweiser et al. [41]. Correspondingly, there were no adverse effects detected on stream biota. In this study, stream temperature, nutrient concentrations, and TSS and turbidity levels did not respond to treatment implementation. As a result, the lack of response of macroinvertebrate metrics to conifer removal treatments is not surprising.

### 3.5: Soil Moisture Tension

The statistical analysis results show that the difference between soil moisture tension in treatment and reference transects increased significantly at both depths in response to the Bailey Project (P < 0.001; n = 725 and 690 at the 15 and 45 cm depths, respectively) and in response to Phase 2 (P < 0.001; n = 666 and 662 at the 15 and 45 cm depths, respectively) and Phase 3 (P < 0.001; n = 677 and 678 at the 15 and 45 cm depths, respectively) of the Pine-Bogard Project. In response to Phase 2 conifer removal, the difference between treatment and reference soil moisture tension increased by 48 and 38 kPa at the 15 and 45 cm depths, respectively. Similarly, in response to Phase 3 conifer removal, the difference between treatment and reference soil moisture tension increased by 31 and 87 kPa at the 15 and 45 cm depths, respectively. Lastly, in response to Bailey Project conifer removal, the difference between treatment and reference soil moisture tension increased by 47 and 67 kPa at the 15 and 45 cm depths, respectively.

**Figure 8:**
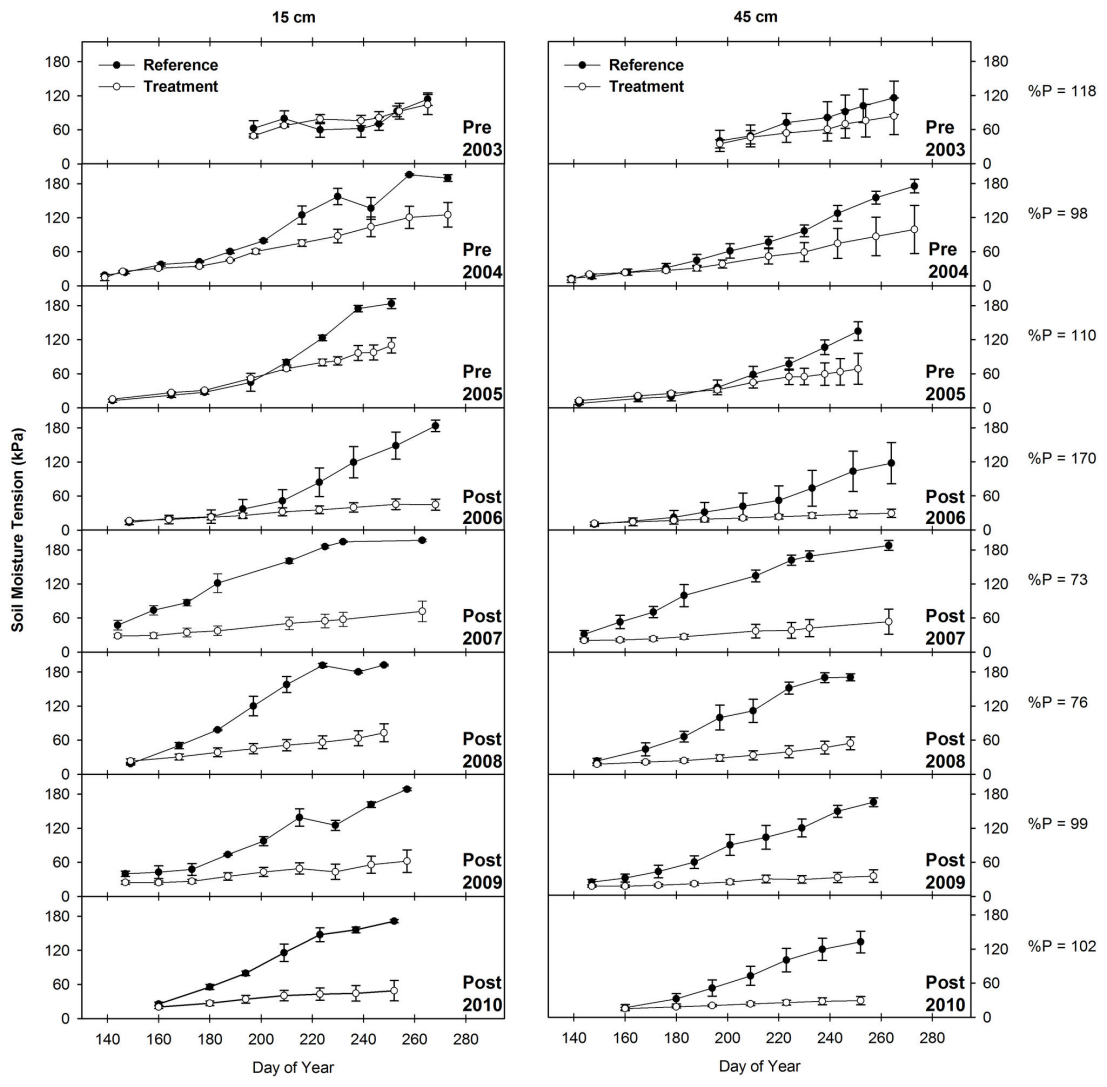
Mean and standard error of soil moisture within the Pine-Bogard Phase 2 August 2005 treatment area. Measurements were made at the 15 and 45 cm depths at Stations C and D of the reference and treatment transects. % P = percent of mean annual precipitation. Lower values of soil moisture tension correspond to wetter soils.

**Figure 9:**
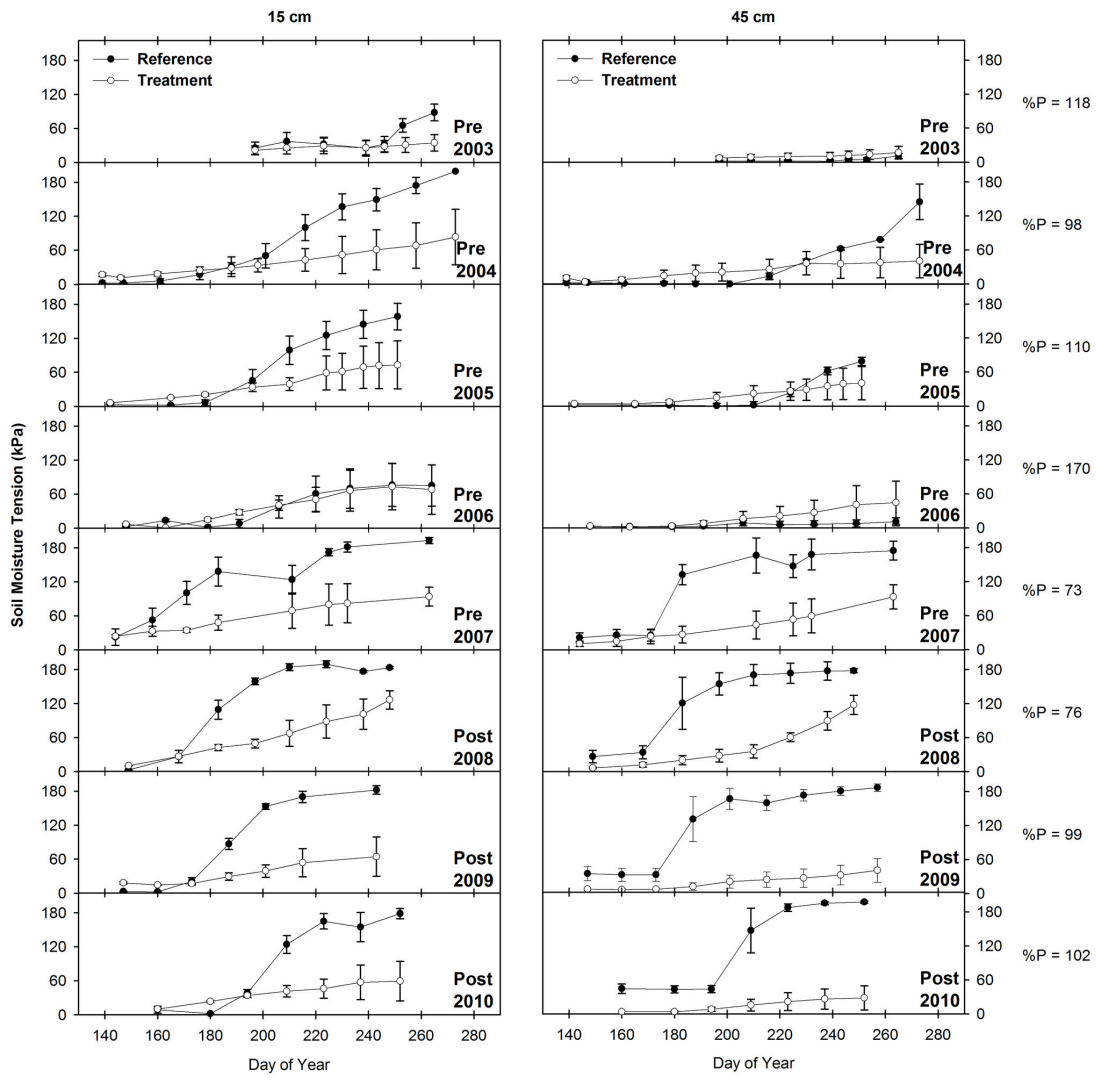
Mean and standard error of soil moisture within the Pine-Bogard Phase 3 January 2008 treatment area. Measurements were made at the 15 and 45 cm depths at Stations A and B of the reference and treatment transects. % P = percent of mean annual precipitation. Lower values of soil moisture tension correspond to wetter soils.

**Figure 10:**
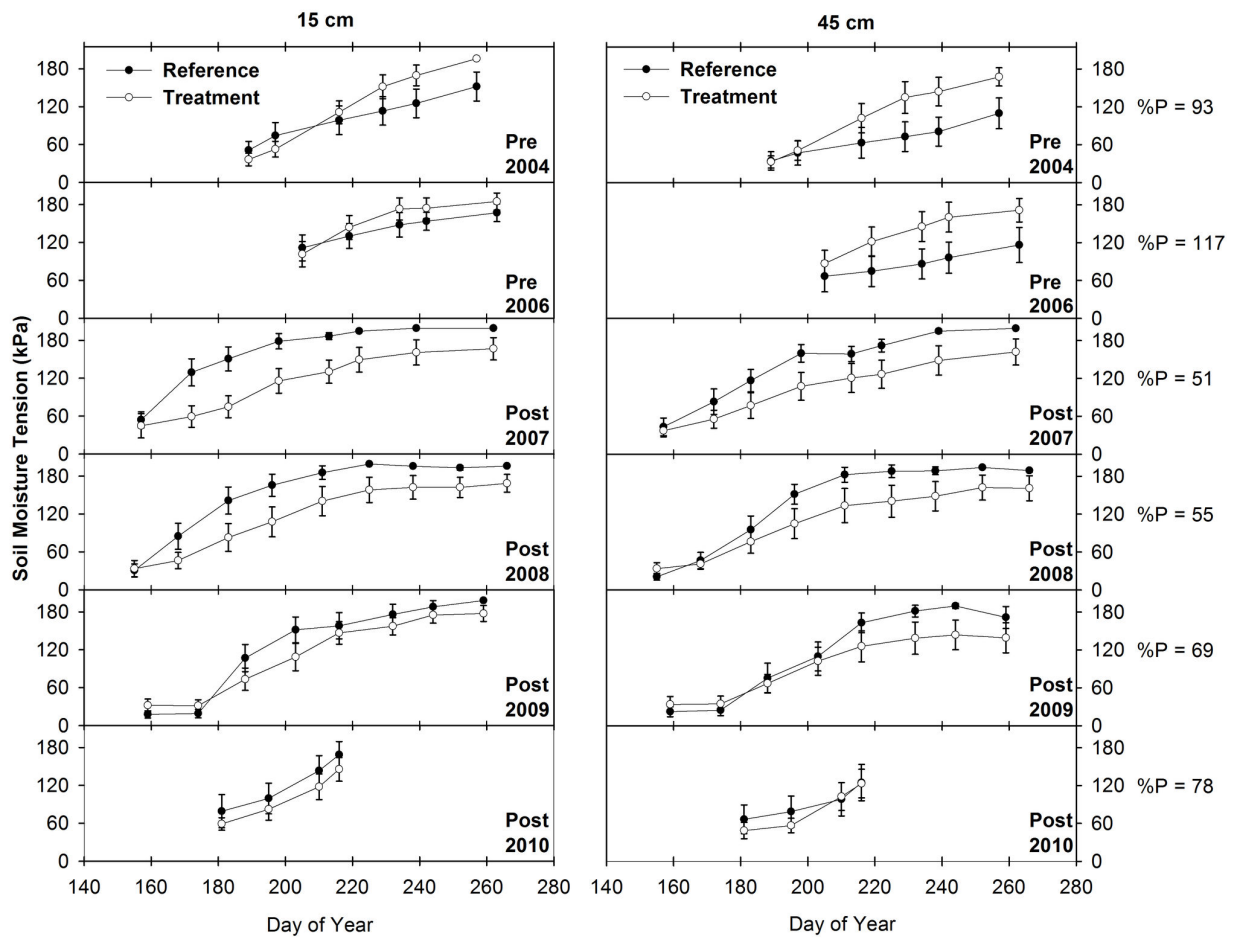
Mean and standard error of soil moisture at the Bailey Creek reference and treatment transects. Measurements were made at the 15 and 45 cm depths. % P = percent of mean annual precipitation. Lower values of soil moisture tension correspond to wetter soils.

The soil moisture tension data illustrated in Figures 8-10 corroborates the results of the statistical analysis and shows that soil moisture in treatment transects increased relative to soil moisture in reference transects at both soil depths. At the Pine-Bogard location, soil moisture tension at both depths was lower (i.e. soil moisture was higher) in the treatment transects than in the reference transects throughout the course of this study (Figures 8 and 9). However, the reference transects exhibited a higher rate of drying relative to treatment transects following both Phase 2 and Phase 3 conifer removal. This trend is particularly evident in the lowest precipitation years from 2007 through 2009 following Phase 2 treatment (Figure 8). During this period, soil moisture tension in reference transects increased (i.e. soil moisture decreased) at a rapid rate relative to previous years, while soil moisture tension in treatment transects showed relatively little response to the drought conditions. At Bailey Creek, soil moisture tension at both depths was higher (i.e. soil moisture was lower) in the treatment transects than in the reference transects prior to treatment implementation (Figure 10). Following conifer removal in September 2006 however, soil moisture tension was lower (i.e. soil moisture was higher) in the treatment transects than in the reference transects. 

The greater retention of soil moisture within treatment transects is most likely the result of the removal of vegetation causing a reduction in transpiration [83], which sustains high soil moisture levels into the dry season. Additionally, the increase in soil moisture could be the result of a decrease in snow sublimation and increase in snow water equivalent (i.e. snow accumulation) that occurs when forest canopy cover decreases [100-103]. Increases in soil moisture in response to timber harvesting have been observed in multiple studies, though, in the long-term, soil moisture levels decrease as vegetation regrows [25,26]. Within conifer-encroached aspen stands however, high soil moisture levels have the potential to be sustained if the site is successfully recolonized by aspen, because mature conifer forests use more water than mature aspen forests [8,104] and because, as a result of higher canopy interception by confers, conifer forests have lower snow water equivalent than deciduous forests [8,103,105,106]. Elevated soil moisture relative to conifer stands is one of the potential factors causing high diversity and productivity in the herbaceous understory of aspen stands [2,107].

## Conclusions

The results of this study suggest that, with careful consideration of site specific conditions and implementation of appropriate best management practices, conifer removal through commercial timber harvesting for the purposes of restoring aspen stands in the southern Cascades can be implemented without degrading the aquatic ecosystem parameters measured. More than 80 % of all stream water samples analyzed for NO_3_-N, NH_4_-N, and PO_4_-P at Pine, Bogard, and Bailey Creeks were below the detection limit, with the exception of PO_4_-P in Bogard Creek, in which concentrations were elevated due to the spring source of the streamwater. There was no significant increase in the difference between upstream and downstream turbidity, TSS, NO_3_-N, NH_4_-N, PO_4_-P, K, and SO_4_-S levels before versus after treatment. There was a significant decrease in canopy cover and increase in the potential fraction of solar radiation reaching the creeks in response to the Pine-Bogard Phase 3 and Bailey treatments; however, there was no corresponding increase in the difference between upstream and downstream temperatures. Macroinvertebrate metrics confirmed the water quality results, with the highest level of % Tolerant species being 0.2 % at Pine Creek prior to treatment implementation. Lastly, soil moisture tension measurements indicate that there was a significant increase in soil moisture in treated aspen stands relative to untreated stands. Although a large body of literature exists in which timber harvest activities were found to impair water quality and aquatic ecosystem functions, the findings of this study concur with recent studies in which the partial harvesting of areas near streams and rivers with the implementation of BMPs was conducted without the degradation of aquatic ecosystems. 

## Supporting Information

Figure S1 **Mean and standard error of turbidity levels for Pine Creek sample stations (2003-2010).** Q = mean annual discharge (m^3^ min^-1^) measured from June 15 through August 31. P = percent of mean annual precipitation.
(PDF)

Figure S2 **Mean and standard error of turbidity levels for Bogard Creek sample stations (2003-2010).** Q = mean annual discharge (m^3^ min^-1^) measured from June 15 through August 31. P = percent of mean annual precipitation.
(PDF)

Figure S3 **Mean and standard error of turbidity levels for Bailey Creek sample stations (2003-2004, 2006-2010).** Q = mean annual discharge (m^3^ min^-1^) measured from June 15 through August 31. P = percent of mean annual precipitation.
(PDF)

Figure S4 **Mean and standard error of total suspended sediment (TSS) concentrations for Pine Creek sample stations (2003-2010).** Q = mean annual discharge (m^3^ min^-1^) measured from June 15 through August 31. P = percent of mean annual precipitation.
(PDF)

Figure S5 **Mean and standard error of total suspended sediment (TSS) concentrations for Bogard Creek sample stations (2003-2010).** Q = mean annual discharge (m^3^ min^-1^) measured from June 15 through August 31. P = percent of mean annual precipitation.
(PDF)

Figure S6 **Mean and standard error of total suspended sediment (TSS) concentrations for Bailey Creek sample stations (2003-2004, 2006-2010).** Q = mean annual discharge (m^3^ min^-1^) measured from June 15 through August 31. P = percent of mean annual precipitation.
(PDF)

Figure S7 **Mean and standard error of NO_3_-N concentrations for Pine Creek sample stations (2003-2010).** Q = mean annual discharge (m^3^ min^-1^) measured from June 15 through August 31. P = percent of mean annual precipitation.
(PDF)

Figure S8 **Mean and standard error of NO_3_-N concentrations for Bogard Creek sample stations (2003-2010).** Q = mean annual discharge (m^3^ min^-1^) measured from June 15 through August 31. P = percent of mean annual precipitation.
(PDF)

Figure S9 **Mean and standard error of NO_3_-N concentrations for Bailey Creek sample stations (2003-2004, 2006-2010).** Q = mean annual discharge (m^3^ min^-1^) measured from June 15 through August 31. P = percent of mean annual precipitation.
(PDF)

Figure S10 **Mean and standard error of NH_4_-N concentrations for Pine Creek sample stations (2003-2010).** Q = mean annual discharge (m^3^ min^-1^) measured from June 15 through August 31. P = percent of mean annual precipitation.
(PDF)

Figure S11 **Mean and standard error of NH_4_-N concentrations for Bogard Creek sample stations (2003-2010).** Q = mean annual discharge (m^3^ min^-1^) measured from June 15 through August 31. P = percent of mean annual precipitation.
(PDF)

Figure S12 **Mean and standard error of NH_4_-N concentrations for Bailey Creek sample stations (2003-2004, 2006-2010).** Q = mean annual discharge (m^3^ min^-1^) measured from June 15 through August 31. P = percent of mean annual precipitation.
(PDF)

Figure S13 **Mean and standard error of PO_4_-P concentrations for Pine Creek sample stations (2003-2010).** Q = mean annual discharge (m^3^ min^-1^) measured from June 15 through August 31. P = percent of mean annual precipitation.
(PDF)

Figure S14 **Mean and standard error of PO_4_-P concentrations for Bogard Creek sample stations (2003-2010).** Q = mean annual discharge (m^3^ min^-1^) measured from June 15 through August 31. P = percent of mean annual precipitation.
(PDF)

Figure S15 **Mean and standard error of PO_4_-P concentrations for Bailey Creek sample stations (2003-2004, 2006-2010).** Q = mean annual discharge (m^3^ min^-1^) measured from June 15 through August 31. P = percent of mean annual precipitation.
(PDF)

Photo S1 **Pine-Bogard Project Phase 1 treatment area (pre-treatment).** Photo taken in 2003.
(PDF)

Photo S2 **Pine-Bogard Project Phase 1 treatment area (post-treatment).** Photo taken in 2004, during the first summer after treatment.
(PDF)

Photo S3 **Pine-Bogard Project Phase 1 treatment area (post-treatment).** Photo taken in 2007, 4 years after treatment.
(PDF)

Photo S4 **Pine-Bogard Project Phase 2 treatment area.** Right side of photo illustrates untreated conifer density, and left side of photo illustrates post-treatment conditions. Photo taken September 2005, immediately following treatment implementation.
(PDF)

Photo S5 **Pine-Bogard Project Phase 3 treatment area prior to conifer thinning.** All blue marked trees were removed, as we all small unmarked trees (< 30 cm DBH). The green of Pine Creek’s riparian area can be seen directly behind the conifer trees marked for removal. Photo taken in 2005.
(PDF)

Photo S6 **Pine-Bogard Project Phase 3 treatment area (post-treatment).** Photo taken in 2008, during the first summer after treatment.
(PDF)

Photo S7 **Pine-Bogard Project Phase 3 treatment area (post-treatment).** Photo taken in 2013, 5 years after treatment.
(PDF)

Photo S8 **Bailey Project (pre-treatment).** Photo taken in 2003.
(PDF)

Photo S9 **Bailey Project (post-treatment).** Photo taken in 2008, 2 years after treatment.
(PDF)

Photo S10 **Bailey Project (post-treatment).** Photo taken in 2011, 5 years after treatment.
(PDF)
