# Supplementary material for: Aquatic Ecosystem Response to Timber Harvesting for the Purpose of Restoring Aspen
Source: PLoS One. 2013 Dec 20;8(12):e84561. doi: 10.1371/journal.pone.0084561 (PMC3869891; doi:10.1371/journal.pone.0084561)

Figure S3. Mean and standard error of turbidity levels for Bailey Creek sample stations (2003-2004, 2006-2010). Q = mean annual discharge ($m^3\ min^{-1}$) measured from June 15 through August 31. P = percent of mean annual precipitation.

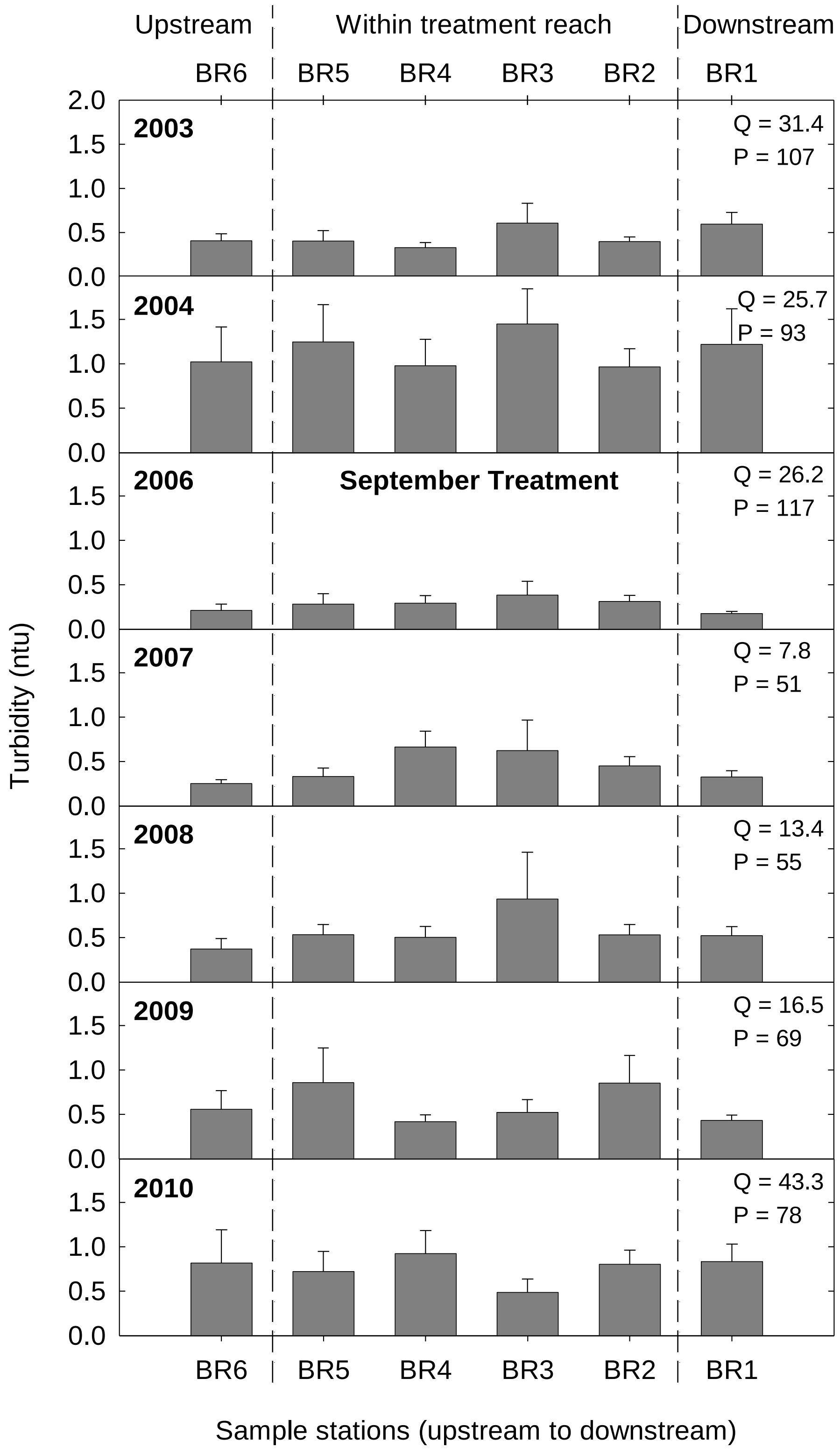

Supplement: Figure S3 — Mean and standard error of turbidity levels for Bailey Creek sample stations (2003-2004, 2006-2010). Q = mean annual discharge (m3 min-1) measured from June 15 through August 31. P = percent of mean annual precipitation. (PDF) [file pone.0084561.s003.pdf]
