# Supplementary material for: Aquatic Ecosystem Response to Timber Harvesting for the Purpose of Restoring Aspen
Source: PLoS One. 2013 Dec 20;8(12):e84561. doi: 10.1371/journal.pone.0084561 (PMC3869891; doi:10.1371/journal.pone.0084561)

Figure S15. Mean and standard error of PO_4-P concentrations for Bailey Creek sample stations (2003-2004, 2006-2010). Q = mean annual discharge (m^3 min^{-1}) measured from June 15 through August 31. P = percent of mean annual precipitation.

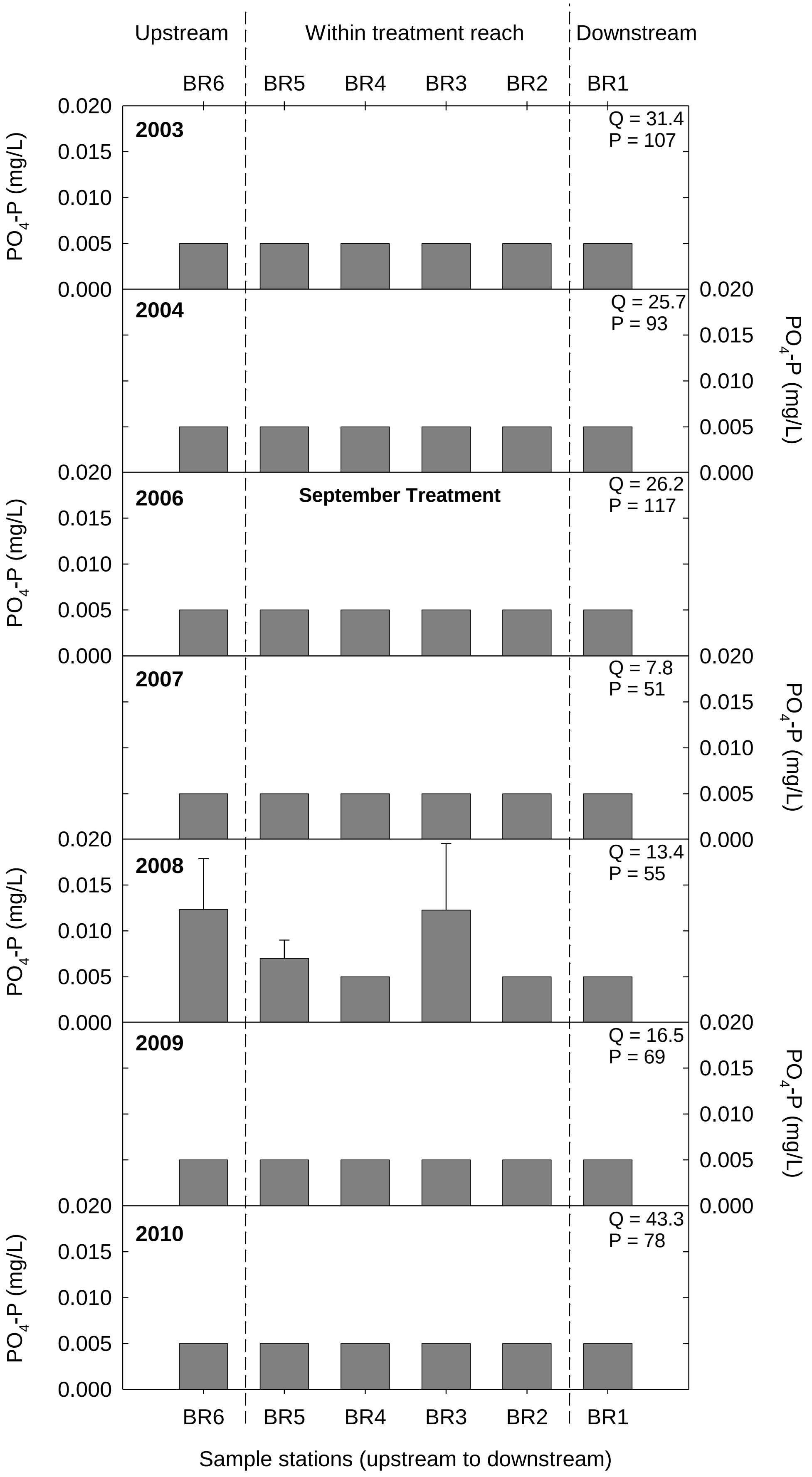

Supplement: Figure S15 — Mean and standard error of PO4-P concentrations for Bailey Creek sample stations (2003-2004, 2006-2010). Q = mean annual discharge (m3 min-1) measured from June 15 through August 31. P = percent of mean annual precipitation. (PDF) [file pone.0084561.s015.pdf]
