# Supplementary figures and images for: Aquatic Ecosystem Response to Timber Harvesting for the Purpose of Restoring Aspen
Source: PLoS One. 2013 Dec 20;8(12):e84561. doi: 10.1371/journal.pone.0084561 (PMC3869891; doi:10.1371/journal.pone.0084561)

Photo S1. Pine-Bogard Project Phase 1 treatment area (pre-treatment). Photo taken in 2003.

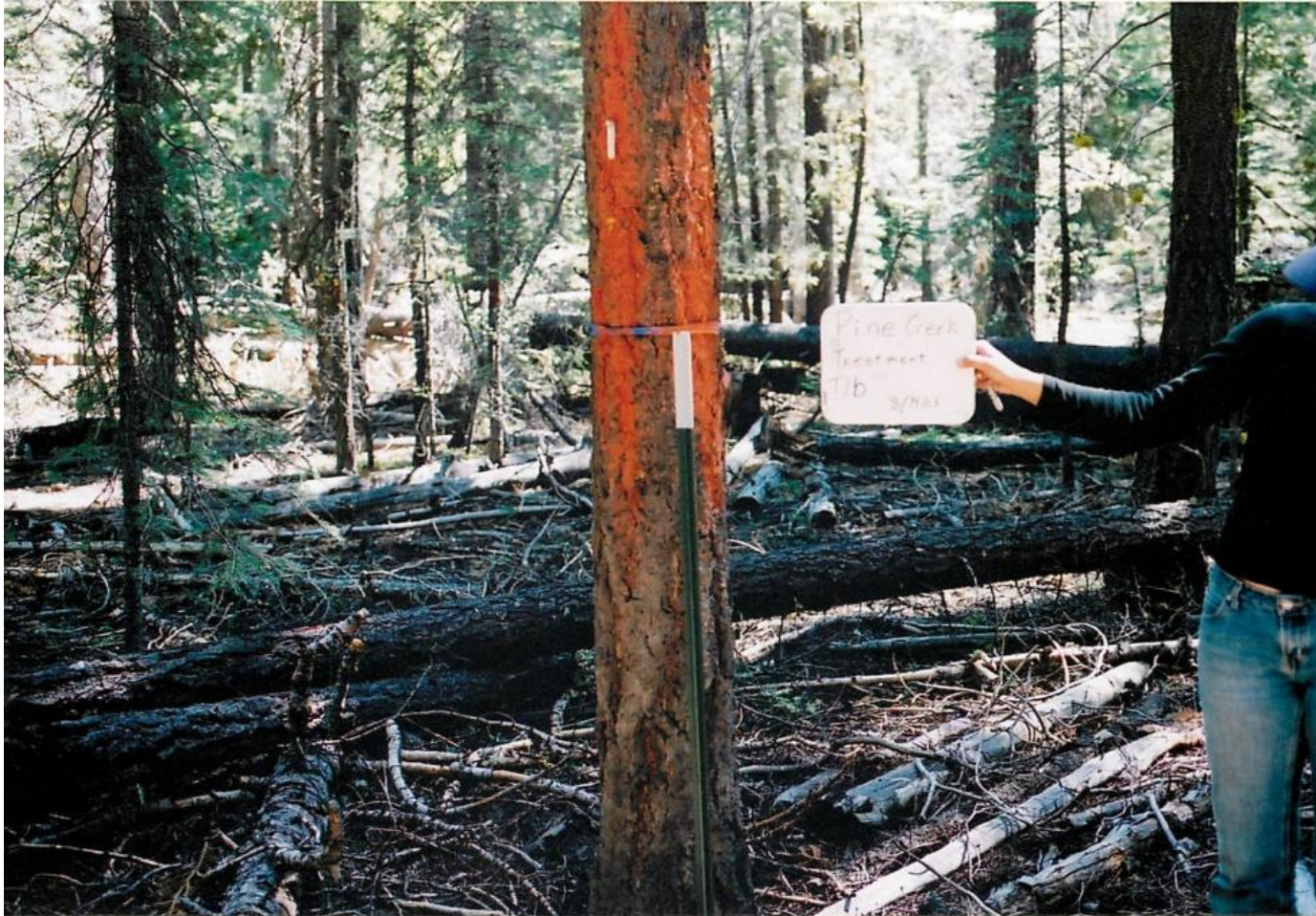

Supplement: Photo S1 — Pine-Bogard Project Phase 1 treatment area (pre-treatment). Photo taken in 2003. (PDF) [file pone.0084561.s016.pdf]

**Photo S8. Bailey Project (pre-treatment).** Photo taken in 2003.

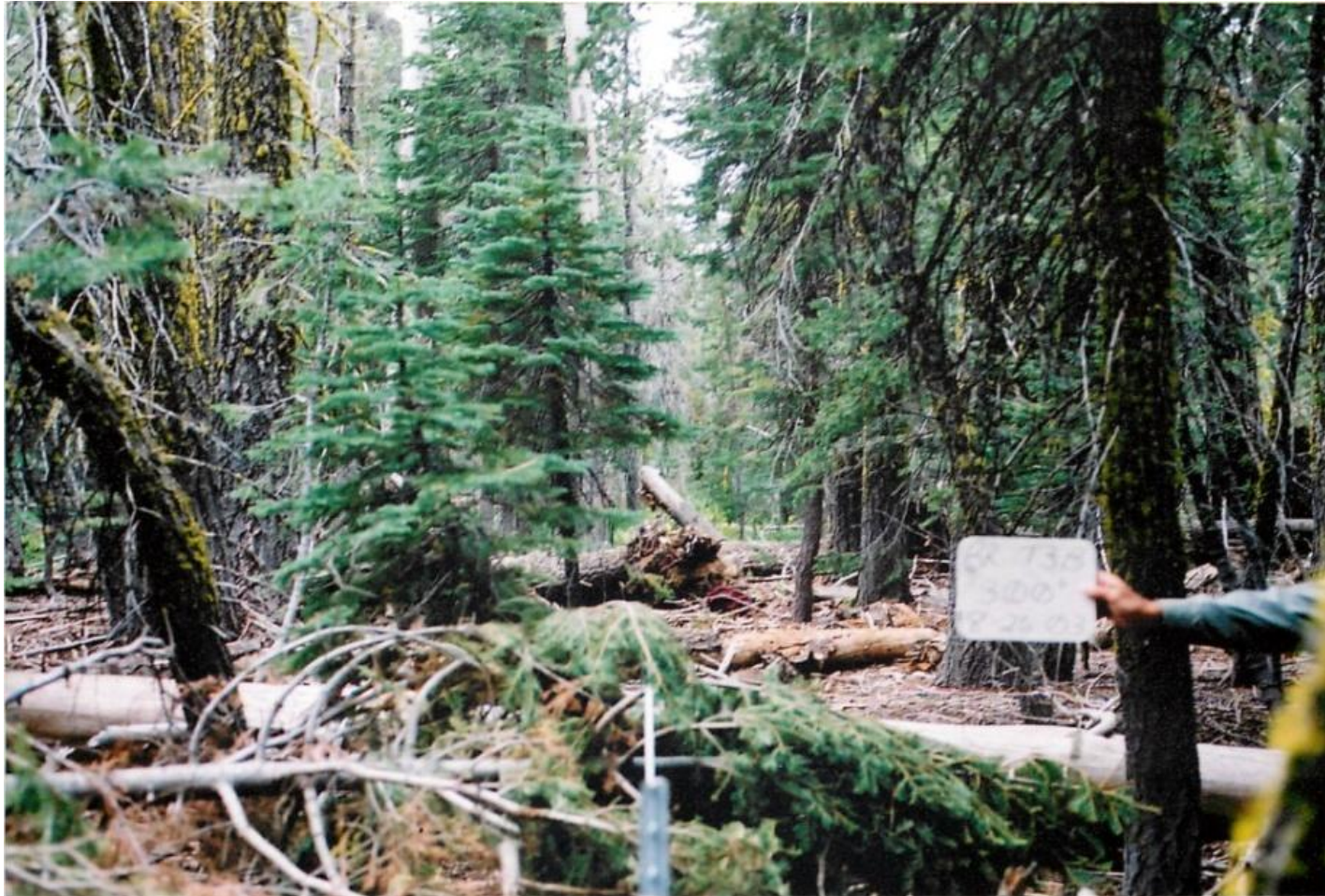

Supplement: Photo S8 — Bailey Project (pre-treatment). Photo taken in 2003. (PDF) [file pone.0084561.s023.pdf]

**Photo S9. Bailey Project (post-treatment).** Photo taken in 2008, 2 years after treatment.

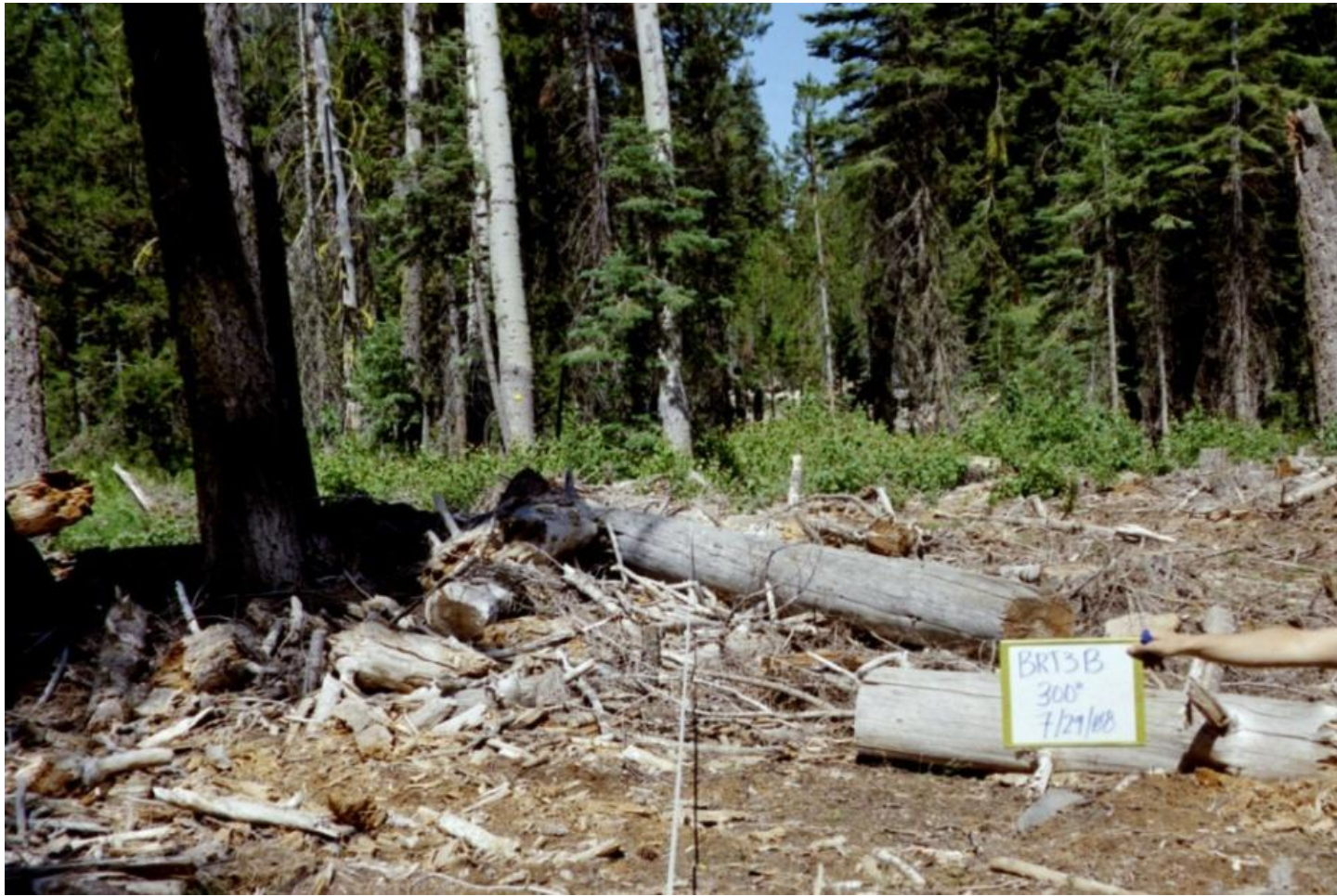

Supplement: Photo S9 — Bailey Project (post-treatment). Photo taken in 2008, 2 years after treatment. (PDF) [file pone.0084561.s024.pdf]

**Photo S10. Bailey Project (post-treatment).** Photo taken in 2011, 5 years after treatment.

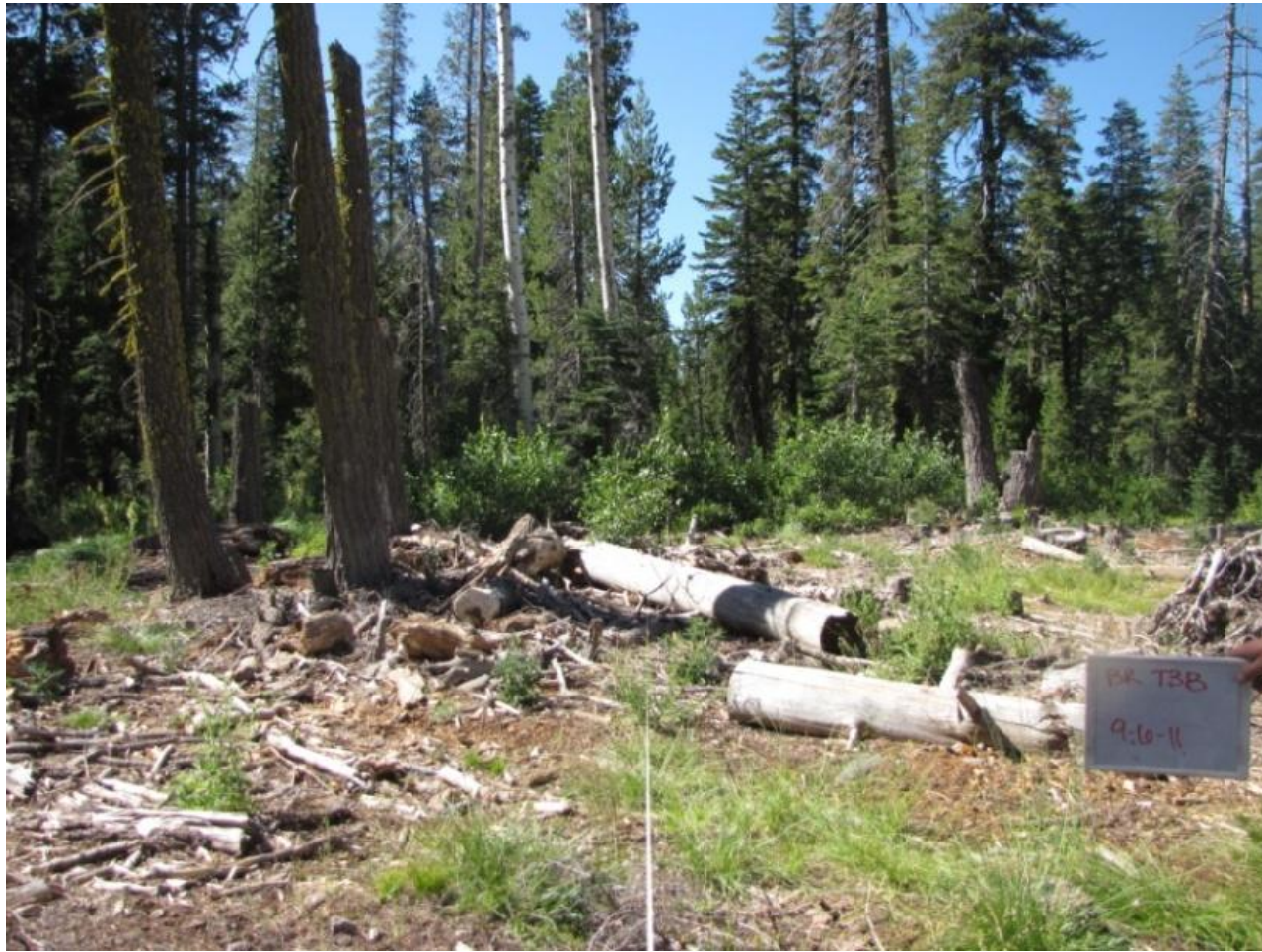

Supplement: Photo S10 — Bailey Project (post-treatment). Photo taken in 2011, 5 years after treatment. (PDF) [file pone.0084561.s025.pdf]
