# Supplementary material for: Aquatic Ecosystem Response to Timber Harvesting for the Purpose of Restoring Aspen
Source: PLoS One. 2013 Dec 20;8(12):e84561. doi: 10.1371/journal.pone.0084561 (PMC3869891; doi:10.1371/journal.pone.0084561)

Photo S4. Pine-Bogard Project Phase 2 treatment area. Right side of photo illustrates untreated conifer density, and left side of photo illustrates post-treatment conditions. Photo taken September 2005, immediately following treatment implementation.

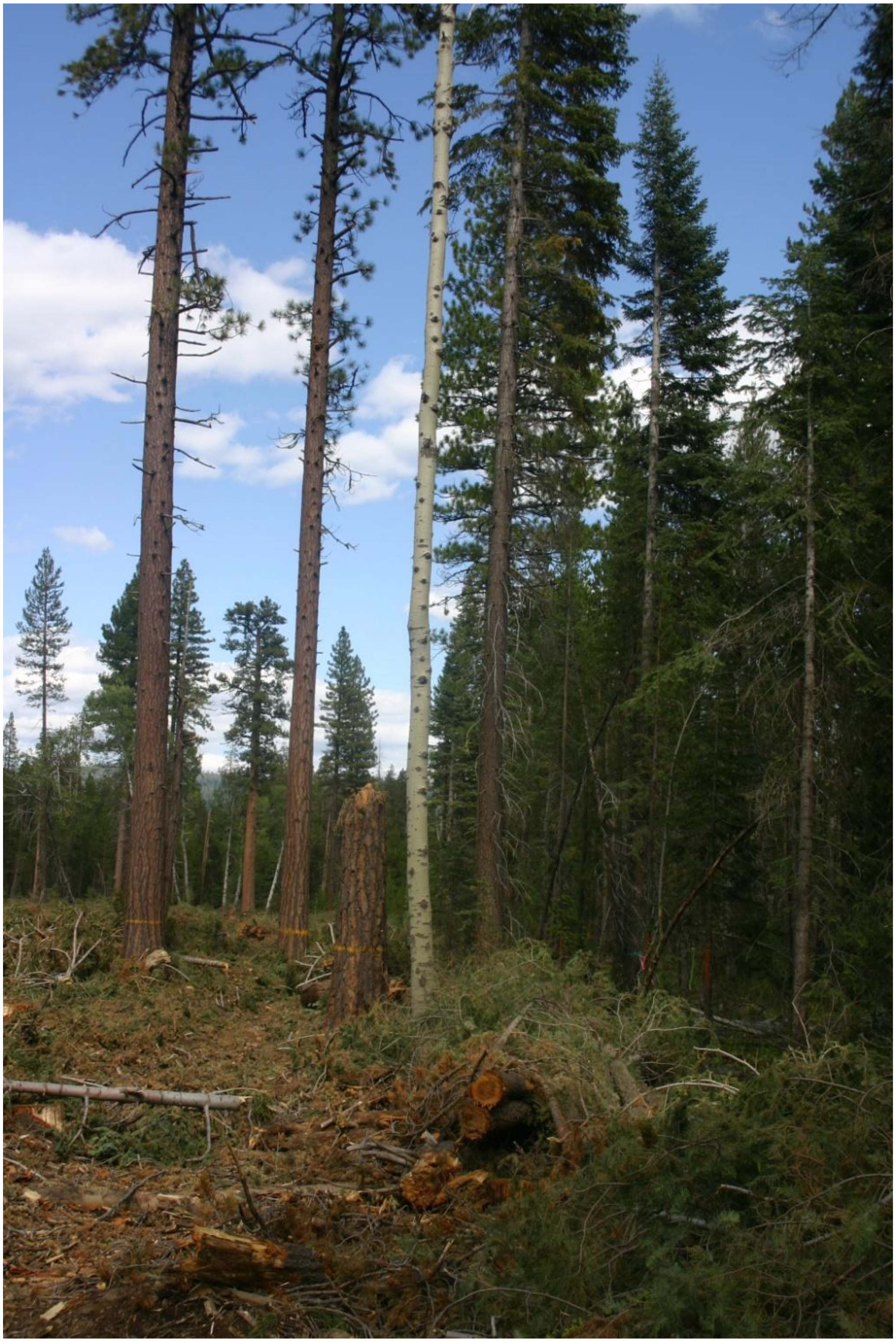

Supplement: Photo S4 — Pine-Bogard Project Phase 2 treatment area. Right side of photo illustrates untreated conifer density, and left side of photo illustrates post-treatment conditions. Photo taken September 2005, immediately following treatment implementation. (PDF) [file pone.0084561.s019.pdf]
