# Supplementary material for: Aquatic Ecosystem Response to Timber Harvesting for the Purpose of Restoring Aspen
Source: PLoS One. 2013 Dec 20;8(12):e84561. doi: 10.1371/journal.pone.0084561 (PMC3869891; doi:10.1371/journal.pone.0084561)

Photo S5. Pine-Bogard Project Phase 3 treatment area prior to conifer thinning. All blue marked trees were removed, as we all small unmarked trees (< 30 cm DBH). The green of Pine Creek's riparian area can be seen directly behind the conifer trees marked for removal. Photo taken in 2005.

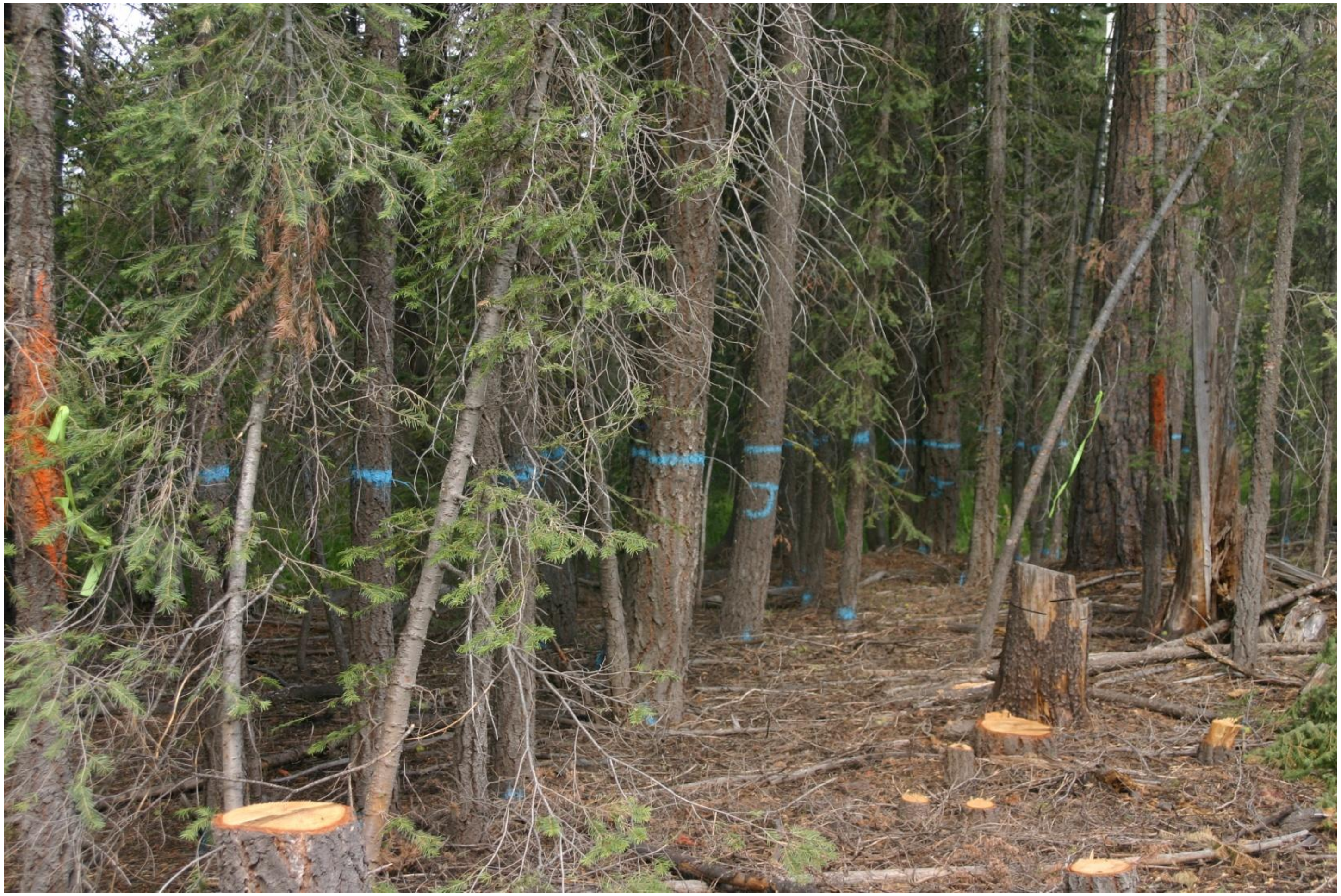

Supplement: Photo S5 — Pine-Bogard Project Phase 3 treatment area prior to conifer thinning. All blue marked trees were removed, as we all small unmarked trees (< 30 cm DBH). The green of Pine Creek’s riparian area can be seen directly behind the conifer trees marked for removal. Photo taken in 2005. (PDF) [file pone.0084561.s020.pdf]
