# Supplementary material for: Aquatic Ecosystem Response to Timber Harvesting for the Purpose of Restoring Aspen
Source: PLoS One. 2013 Dec 20;8(12):e84561. doi: 10.1371/journal.pone.0084561 (PMC3869891; doi:10.1371/journal.pone.0084561)

Photo S7. Pine-Bogard Project Phase 3 treatment area (post-treatment). Photo taken in 2013, 5 years after treatment.

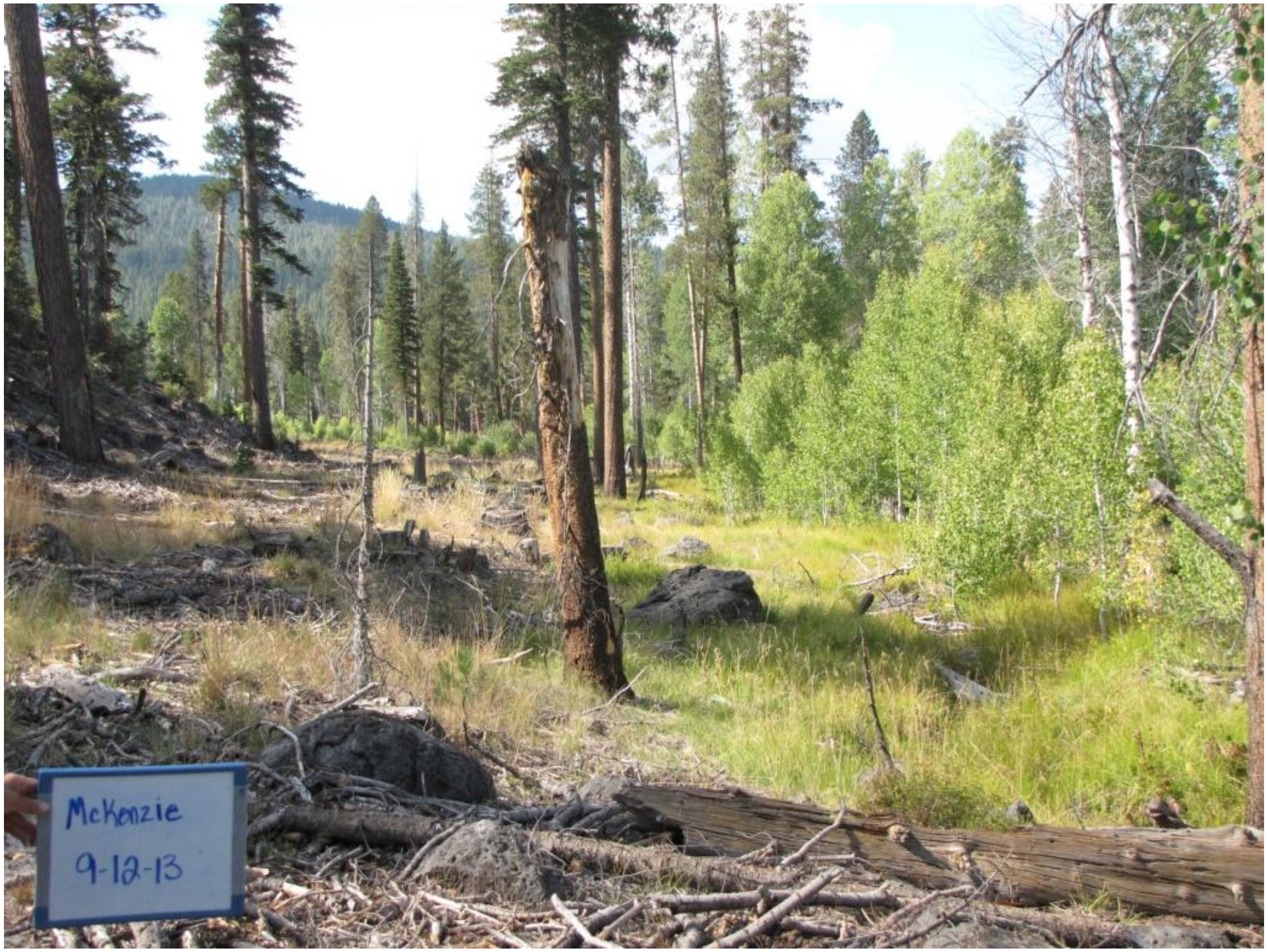

Supplement: Photo S7 — Pine-Bogard Project Phase 3 treatment area (post-treatment). Photo taken in 2013, 5 years after treatment. (PDF) [file pone.0084561.s022.pdf]
